# Unravelling the interplay of ecological processes structuring the bacterial rare biosphere

**DOI:** 10.1038/s43705-022-00177-6

**Published:** 2022-10-03

**Authors:** Xiu Jia, Francisco Dini-Andreote, Joana Falcão Salles

**Affiliations:** 1 Microbial Ecology Cluster, Genomics Research in Ecology and Evolution in Nature (GREEN), Groningen Institute for Evolutionary Life Sciences (GELIFES), University of Groningen, Groningen, 9747AG The Netherlands; 2 Department of Plant Science, The Pennsylvania State University, Pennsylvania, University Park, PA 16801 USA; 3 Huck Institutes of the Life Sciences, The Pennsylvania State University, University Park, PA 16801 USA

**Keywords:** Microbial ecology, Community ecology

## Abstract

Most ecological communities harbor many rare species (i.e., the rare biosphere), however, relatively little is known about how distinct ecological processes structure their existence. Here, we used spatiotemporal data on soil bacterial communities along a natural ecosystem gradient to model the relative influences of assembly processes structuring the rare and common biospheres. We found a greater influence of homogeneous selection (i.e., imposed by spatiotemporally constant variables) mediating the assembly of the rare biosphere, whereas the common biosphere was mostly governed by variable selection (i.e., imposed by spatial and/or temporal fluctuating variables). By partitioning the different types of rarity, we found homogeneous selection to explain the prevalence of permanently rare taxa, thus suggesting their persistence at low abundances to be restrained by physiological traits. Conversely, the dynamics of conditionally rare taxa were mostly structured by variable selection, which aligns with the ability of these taxa to switch between rarity and commonness as responses to environmental spatiotemporal variations. Taken together, our study contributes to the establishment of a link between conceptual and empirical developments in the ecology of the soil microbial rare biosphere. Besides, this study provides a framework to better understand, model, and predict the existence and dynamics of microbial rare biospheres across divergent systems and scales.

## Introduction

Ecological communities are generally composed of a few highly abundant species and numerous low abundance ones, which the latter is defined as the rare biosphere [1, 2]. In microbiomes, rare microbial species potentially play crucial roles in ecosystem functioning, for instance, by preventing pathogen spread, controlling nutrient cycling, and contributing to pollutant degradation [3–6]. However, it remains unclear how distinct ecological processes interplay in determining the assembly and successional dynamics of the different types of microbial rarity.

In plant and animal communities, rarity can be caused by either lower competitive ability combined with frequency-dependent selection (e.g., predation), narrow niche requirement, or limited dispersal ability [7–10]. These mechanisms are likely to apply to microorganisms [11]. For example, protozoan predation and viral lysis can both control population size and occasionally lead to rarity [12]. Also, bacterial specialists can occupy niches that are not explored by generalists, thus being able to persist at low abundances [13]. And, limited dispersal potential was recently used to explain rarity in bacterioplankton taxa [14]. Although these mechanisms are diverse, they are not in conflict and can operate simultaneously. In fact, it is likely that their relative importance might vary in space and/or time and across systems, thus contributing to different abundance patterns of rarity within and across community types.

Distinct ecological processes (i.e., selection, dispersal, diversification, and ecological drift) interplay in structuring ecological communities [15–17]. We have recently argued that understanding the interplay of ecological processes structuring the dynamics of the rare biosphere can better inform on the mechanisms associated with the different abundance patterns of rarity (see ref. [18]). For example, the relative influence of distinct ecological processes can be quantified by comparing the phylogenetic and taxonomic community structures with ecological null model randomizations (see Stegen’s approach in ref. [19, 20]). This approach assumes that phylogenetically related species have more similar ecological niches than those drawn randomly from the phylogenetic tree [21]. After differentiating common from rare species, which can be done using distinct methods and approaches, the first step consists of determining whether the community structure of each one of these components (rare or common) is predominantly driven by selection (either variable or homogeneous) or not (i.e., stochastic assembly) [18, 22]. This information can be obtained by comparing the phylogenetic structure of observed communities with the distribution of the phylogenetic community structures of null models (Supplementary Fig. 1, Step 1). In brief, null models are built based on randomization of phylogenetic community structures. When the phylogenetic turnover is significantly higher than that of the null expectation, community assembly is primarily structured by variable selection, which indicates that community turnover is regulated by shifting environmental conditions over space or time. However, if the phylogenetic turnover of the community is significantly lower than that of the null expectation, community assembly is mediated by homogeneous selection, suggesting that community turnover is governed by environmental filter(s) that are not undergoing spatiotemporal changes [20, 23]. When the phylogenetic distance between a given pair of communities is not significantly different from the null distribution, selection is weak, thus indicating that community turnover is primarily governed by stochastic processes such as random dispersal and/or ecological drift. These processes can be further disentangled by calculating the dissimilarity of taxonomic composition between two communities [Bray-Curtis (BC) distance] relative to the null distribution, using a Raup-Crick matrix [19, 24] (Supplementary Fig. 1, Step 2). Here the assumptions are the following: (*i*) high dispersal rates lead to homogeneous species distribution across communities (i.e., homogenizing dispersal; observed BC is lower than null BC); (*ii*) low dispersal causes high species turnover (i.e., dispersal limitation; observed BC is higher than null BC); and (*iii*) when both phylogenetic and taxonomic community structures do not differ from the null expectations, neither selection nor dispersal contributes significantly to community assembly. In this scenario, ecological drift and the negligible selection and dispersal effects drive community turnover and are collectively termed ‘undominated processes’ [20].

The approach described above can be used to better understand the ecological processes associated with different types of rarity based on the abundance of taxa across space and/or time. In this sense, the types of rarity consist of ‘conditionally rare,’ ‘permanently rare,’ and ‘transiently rare’ taxa [18, 22]. Conditionally rare species are those that can periodically or occasionally fluctuate from low to high abundance; permanently rare taxa consistently persist at low abundance regardless of environmental changes over space and time; and transiently rare taxa are those that only occur in a community for a short period of time and do not maintain detectable levels of population sizes. The overarching hypothesis of our study is that these different types of rarity are governed by a distinct interplay of ecological processes [18]. Specifically, conditionally rare taxa can be found in the rare biosphere under unfavorable physicochemical or biological conditions but become occasionally or periodically abundant once local conditions become favorable [25]. Therefore, we hypothesize that conditionally rare taxa are driven by variable selection, caused by their fitness changes along environmental (spatial or temporal) gradients [18]. Conversely, permanently rare taxa consistently remain at low abundance regardless of the changing environmental factors [26]. As such, we hypothesize the existence of these taxa to be structured by homogeneous selection [18], as the low abundance and fitness of permanently rare taxa might be associated with k-strategists. For example, specialized oligotrophic bacteria displaying a narrow ecological niche (and/or lower competitive ability) are expected to have minimal response to seasonal fluctuations of the labile carbon pool through the year, as their metabolisms are primarily responding to recalcitrant carbon resources that are more constantly available over time [27]. Permanently rare taxa that show periodic fluctuations in abundance can be determined by homogenizing dispersal process operating alongside ecological drift [18]. This might occur because high dispersal rates replenish the loss of individuals that can occasionally go extinct. For these transiently rare taxa, we hypothesized that dispersal limitation and local extinction through stochastic demographic processes might result in their brief appearance in a community [18]. Finally, the diversification process can also account for new species/genotypes within the rare biosphere (either by mutation and/or horizontal gene transfer) [18]. If this process results in a higher fitness, organisms will persist over time (permanently or conditionally rare); otherwise, they will be either eliminated by natural selection or drift and then constrained as transiently rare taxa [18]. It is important to note that our analysis cannot quantitatively account for the diversification process due to inherent methodological limitations associated with identifying new species/genotypes in natural ecosystems.

In the present study, we empirically quantified the extent to which these ecological processes govern the assembly and successional dynamics of the distinct rarity types (see ref. [18, 28]) and the abundant subcommunities in soil bacterial communities. To test our hypotheses, we used a dataset from an ecological gradient of primary soil succession spanning over a century on the island Schiermonnikoog, the Netherlands, which provides variation in biotic and abiotic conditions while under the influence of the same species pool [28]. The putatively ‘active’ bacterial rare and common biospheres were characterized by sequencing the reverse transcribed bacterial 16S rRNA transcripts obtained from soil bacterial communities [28]. The profiling of the rare biosphere along this gradient at different points in time allowed us to cover great variation of species turnover and identify the distinct types of rarity across both spatial and temporal scales. Moreover, the characterization of the common microbial biosphere allowed us to identify the mechanisms driving the assembly of common species. In this study, we first partitioned the common and rare biosphere components using three rarity cutoff values (i.e., 0.2%, 0.1%, and 0.05%) and applied the null model approach to quantify the relative influence of ecological processes [19, 20]. We focused on the results from the cutoff of 0.1% per sample as it can better represent the rare biosphere by showing the tails of rank abundance curves (Supplementary Fig. 2). As there is still a lack of a golden standard to define the rare biosphere, additional two cutoffs (0.2% and 0.05%) were used to check whether a more relaxed or strict cutoff will influence the interpretation of the results. Then, we categorized and quantified the distinct types of rarity and commonness (Supplementary Fig. 3) [18, 22]. Last, we provide a conceptual overview of how community assembly processes regulate different components of bacterial communities, which will serve as the basis for further hypotheses development in this emergent field of science.

## Materials and methods

### Soil sampling and sequencing

**Fig. 1:**
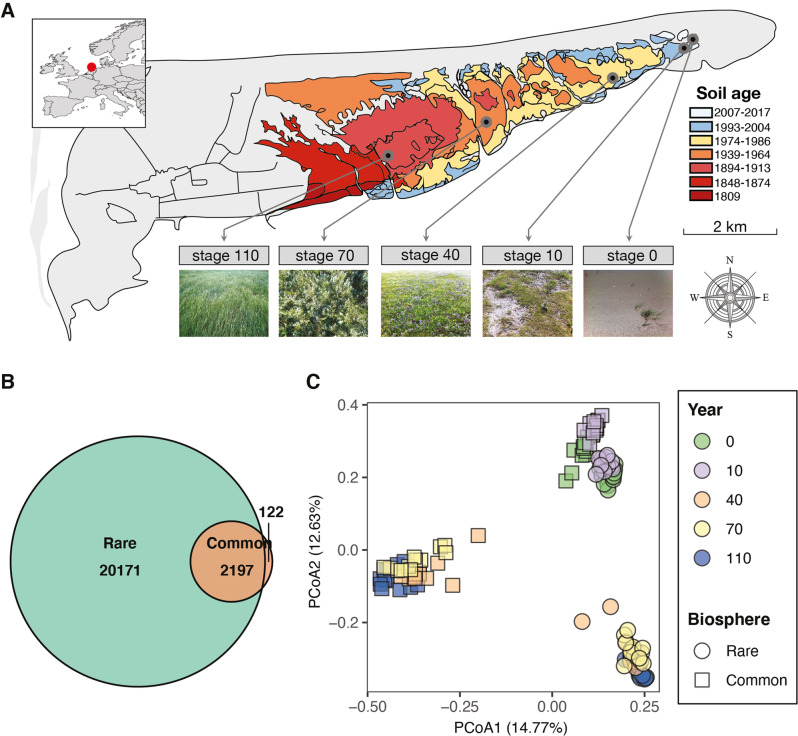
Distribution of soil bacterial common and rare biospheres. **A** Sampling sites are shown on the map of the island of Schiermonnikoog, the Netherlands. Distinct colors represent different successional stages along the soil chronosequence that gradually develops from the east to the west. Dots represent the sampling sites at five successional stages, namely 0, 10, 40, 70, and 110 years of succession (from 1809 to 2017). Modified version from Dini-Andreote et al. [79]. This image was originally generated using ArcGIS. **B** The number of rare and common ASVs and the number of shared ASVs in the rare and common biospheres. **C** Principal Coordinate Analysis (PCoA) derived from Bray-Curtis distance of bacterial composition for the rare and common biospheres represented by different shapes. Colors represent successional stages (0, 10, 40, 70, and 110 years in succession). Percentage in the axis labels shows the variation of species composition explained by each coordinate.

In this study, we used the 16S rRNA sequence dataset from [28], which has been deposited at the Sequence Read Archive of the National Center for Biotechnology Information and is accessible through the accession number PRJNA546612. Details of soil sampling and sequencing are provided in SI Methods and ref. [28]. In brief, soil samples were collected along a well-characterized soil chronosequence located on the island of Schiermonnikoog, the Netherlands (53°30’ N, 6°10’ E). This chronosequence covers over 100-years of primary succession in a developing salt marsh ecosystem since the sedimentation of particles carried out by the tide/wind causes the island to progressively extend eastwards [29]. Soil physicochemical properties and community composition (both macro- and micro-organisms) sequentially change over time along with the gradient [29–32]. For example, this chronosequence presents a transition from sandy to clay soil. In addition, the overall soil nutrient status (i.e., organic carbon content, total nitrogen, ammonium, nitrate, and sulfate) increases over time, whereas soil pH decreases from ca. 8.7 to 7.4, as the succession proceeds [30]. We collected soil samples across five successional stages (i.e., 0, 10, 40, 70, and 110 years of development from 1809 to 2017) to capture the variation in the rare biosphere across these sites (Fig. 1A). Three replicated plots were sampled in each successional stage. In each plot, one composite soil sample was obtained (details see ref. [28]). Sampling was performed in May, July, September, and November 2017 to include the temporal dynamics within each site. In total, we analyzed the assembly of the bacterial rare biosphere from 60 soil samples. To capture the putatively ‘active’ bacteria from soil, the V4 region of bacterial 16S rRNA was amplified based on reverse-transcribed total soil RNA using the primer set 515 F and 806 R [33, 34]. Sequencing was performed on a 151 bp × 12 bp × 151 bp Illumina MiSeq run (Illumina, USA) at the Argonne National Laboratory using the Version 2 chemistry sequencing reagent kit [33].

We opted for an RNA-based approach to profile the bacterial rare biosphere rather than the usual DNA-based method. The latter has the risk of misestimating rare species due to the pervasive existence of relic DNA in soil [35]. Although RNA-based approaches also have limitations, such as a variable number of ribosomal transcripts at different activities, we argue that this approach is relevant for rare biosphere studies, as it controls for pseudo-rare species that can be potentially detected by DNA-based methods. Even though the RNA approach might wrongly classify rare species with high copy numbers of rRNA as abundant, it provides certainty that all active rare species are real and from alive cells. Since the overall assembly processes did not differ significantly between DNA- and RNA-based approaches [28], we expected to draw similar conclusions for the rare biosphere when using the DNA-based approach.

### Sequence processing

Sequence data analysis was performed using the open-source QIIME2/2018.2 pipeline [36]. Samples were demultiplexed, resulting in a total of 9 852 975 raw reads across the 60 samples. We applied the Divisive Amplicon Denoising Algorithm (DADA2) to infer exact/amplicon sequence variants (ASVs) [37]. The DADA2 method was applied to denoise paired-end sequences trimmed at 150 bp, paired, and subjected to chimera removal using the default settings. The obtained feature table (site-to-species matrix) contained a total of 24 172 ASVs. The total frequency of these ASVs across all samples was 7 958 654. Taxonomic information for the representative sequence per ASV (ca. 253 bp in length) was obtained using the SILVA database (Silva 119 Naive Bayes 515 F/806 R taxonomy classifier) [38]. A phylogenetic tree was constructed by aligning representative sequences per ASV using the FastTree plugin in QIIME2 [39].

### Defining the rare and common biospheres

After compiling the feature and taxonomy tables, community analyses and statistics were performed in the R environment (R version 3.5.0) [40, 41]. Figures were generated using the ggplot2 and venn.diagram packages [42, 43]. The feature and taxonomy tables were combined, and all ASVs affiliated with archaea, chloroplasts, and mitochondria were removed. The feature table was rarefied to a depth of 31 500 sequences per sample using the ‘rarefy’ function in the package vegan [44]. The rarefied feature table was used for downstream analyses.

To depict sample-by-sample variation in rarity, we defined rarity on a per sample basis rather than based on the entire dataset. In particular, the rare biosphere was defined as a collection of low abundance taxa with relative abundance no greater than the rarity cutoff values. In contrast, species with relative abundance higher than the cutoffs were collectively defined as the common biosphere. We first fitted the frequently used rarity cutoffs, i.e., 1.0%, 0.1%, and 0.01% [45–49], to the rank abundance curves in our dataset (Supplementary Fig. 2B) to determine the most suitable cutoff value. These plots show that using 1.0% or 0.01% would either overestimate or underestimate the size of the rare biosphere in our samples (Supplementary Fig. 2B). In addition, to complement this fixed threshold, we applied a ‘sample-specific rarity cutoff’ approach, in which rarity is defined using a similar algorithm to calculate the h-index [50], where species are defined as rare when their abundances are not higher than their individual ranks in the rank abundance curve (Supplementary Figs. 4 and 5). We recalibrated the cutoff by the sampling depth value, i.e., the ratio of the number of observed species (S_obs_) to the number of estimated species (S_chao1_), to balance the difference in sampling effort between samples. Here we used the Chao1 index to indicate estimated richness. It is worth noticing that the estimated species number (Chao1) can be higher than the true species richness, as it can overestimate species numbers when the sample size is small [51]. However, since our rarefaction curves seem to have reached a plateau steadily, and the observed number of species was close to the estimated species by the Chao1 index in most samples (Supplementary Figs. 6 and 7), we believe the Chao1 index did not overestimate richness substantially in our dataset. However, the use of this index in future studies should be carefully evaluated. Using the “sample-specific rarity cutoff” approach, we identified the average of sample-specific rarity cutoffs at 0.2% (Supplementary Figs. 2A and 5). Furthermore, to avoid the arbitrary definition of the rare biosphere, we tested two additional rarity cutoffs, i.e., 0.2% and 0.05%, which aligned with the tails of the relative abundance curves and represented interval deviations from the usual fixed threshold of 0.1%.

We divided the dataset into two community components (the rare and common biospheres) using the criteria described above, which entailed the tails of rank abundance curves in our dataset (0.2%, 0.1%, and 0.05%; Supplementary Fig. 2A). We mainly show the results based on the cutoff of 0.1% and briefly discuss whether a more relaxed (0.2%) or strict cutoff (0.05%) provide similar results as the cutoff of 0.1%.

### Defining the distinct types of rarity and commonness

After defining the rare and common biospheres, we further classified rare and common ASVs into distinct types of rarity and commonness (Supplementary Fig. 3), as follows: ‘conditionally rare/common’—rare ASVs in one or few samples that are occasionally common in other samples; ‘transiently rare’—ASVs that appear only once in the rare biosphere across all samples (stage of succession and sampling time); ‘permanently rare’—ASVs that are only present in the rare biosphere and appear more than once across all the samples; ‘permanently common’—ASVs consistently present above the rare threshold across all samples. It should be mentioned that properly defining permanently rare ASVs is challenging. In this study, we sampled bacterial communities across a spatial chronosequence (from early ‘sandy’ to mature ‘clay’ soils), considering within-stage temporal variations (from May, July, September, and November). Such efforts allowed a thorough depiction of soil bacterial communities in this system, thus supporting a valid representation of the distinct types of rarity, particularly with respect to permanently rare ASVs.

### Community analysis

Principal coordinate analysis (PCoA, ‘pcoa’ function in the package ape) was used to explore and visualize community dissimilarities using Bray-Curtis distances (‘vegdist’ function in the vegan package) [44, 52]. To assess whether biosphere component, stage of succession, and sampling time had significant effects on the bacterial community structure, permutational multivariate analysis of variance (PERMANOVA, ‘adonis’ function in the vegan package) was performed based on Bray-Curtis distances using 9 999 random permutations.

### Quantifying the relative influences of distinct community assembly processes

To quantify community assembly processes structuring the common and rare biosphere, we used a previously developed approach [19]. Namely, phylogenetic community turnover was inferred based on the β-nearest taxon index (βNTI) across all samples. We calculated βNTI using the package ‘Picante’ and the script created by Stegen et al. [19, 53], which is the standardization of between-community mean nearest taxon distance (βMNTD), as follows:

$$\beta {{{{{{{\mathrm{MNTD}}}}}}}} = 0.5\left[ {\mathop {\sum}\nolimits_{i_k = 1}^{n_k} {f_{ik}\min \left( {\Delta i_kj_m} \right)} + \mathop {\sum}\nolimits_{i_m = 1}^{n_m} {f_{im}\min \left( {\Delta i_mj_k} \right)} } \right],$$

$$\beta N{{{{{{{\mathrm{TI}}}}}}}} = \frac{{\beta {{{{{{{\mathrm{MNTD}}}}}}}}_{obs} - \overline {\beta {{{{{{{\mathrm{MNTD}}}}}}}}_{null}} }}{{sd\left( {\beta {{{{{{{\mathrm{MNTD}}}}}}}}_{null}} \right)}},$$

where $f_{ik}$ is the relative abundance of ASV *i* in community *k*, *n*_*k*_ is the number of ASVs in community *k*, *f*_*im*_ is the relative abundance of ASV *i* in community *m*, *n*_*m*_ is the number of ASVs in community *m*, and $\min \left( {\Delta i_kj_m} \right)$ is the phylogenetic distance among closest ASVs occurring in community *k* and community *m*. The distribution of βMNTD from null models was built by shuffling ASVs among the tips of the phylogenetic tree with 999 permutations. βNTI is a standardized value of the $\beta {{{{{{{\mathrm{MNTD}}}}}}}}$ of the observed sample ($\beta {{{{{{{\mathrm{MNTD}}}}}}}}_{obs}$) that was scaled by the $\beta {{{{{{{\mathrm{MNTD}}}}}}}}$ of null distribution ($\beta {{{{{{{\mathrm{MNTD}}}}}}}}_{null}$). βNTI values above +2 indicate a higher phylogenetic turnover in observed communities than in the null model distribution. This indicates a strong influence of variable selection on community turnover. βNTI values below −2 indicate lower phylogenetic community turnover in observed communities than in the null model distribution, which further indicates the influence of homogeneous selection on community assembly [19, 20, 23]. If −2 < βNTI < +2, community turnover does not significantly deviate from null expectation, and is thus governed mostly by stochastic processes, such as dispersal limitation, homogenizing dispersal, or undominated processes. In a follow-up analytical step, the Raup-Crick matrix based on the standardized Bray-Curtis matrix (referred to as RC_bray_) was used to test whether the observed degree of turnover deviates from the expectation [24]. We applied the script created by Stegen et al. to compute the RC_bray_ matrix for all communities [19]. A null distribution of the Raup-Crick matrix was built by simulating 999 times for each pair of communities. The RC_bray_ was calculated by the deviation between observed values and the null distribution and rescaled to a range from −1 to +1. RC_bray_ > +0.95 indicates dispersal limitation coupled with drift that leads to community turnover greater than expected, whereas RC_bray_ < −0.95 indicates that community turnover is primarily governed by homogenizing dispersal. Last, −0.95 < RC_bray_ < +0.95 is interpreted as indicating undominated processes. We quantified the relative influences of each of these processes by calculating the percentage βNTI and RC_bray_ values that fulfill the above criteria across all the pairwise comparisons per each community compartment (Supplementary Fig. 1, Step 3).

## Results

### Community profiling

We obtained a rarefied feature table containing 31 500 cDNA sequences per sample, encompassing a total of 22 490 potentially active ASVs. Rarefaction curves of most of the samples reached a steady plateau (Supplementary Fig. 6). As a consequence, the estimated ASVs richness using the Chao1 index was mostly equivalent to the observed richness, indicating a good representation of ASV richness in our dataset (Supplementary Fig. 7). In accordance with previous observations [30], we showed that the structure of bacterial communities significantly changed along the successional chronosequence (PERMANOVA *R*^2^ = 0.45, *P* < 0.01 for Jaccard distance and *R*^2^ = 0.61, *P* < 0.01 for Bray-Curtis distance; Supplementary Fig. 8 and Table 1).

### Bacterial community composition of the rare and common biospheres

Based on a rarity cutoff of ≤0.1% of total relative abundance per community, the richness of the rare biosphere (encompassing 22 368 ASVs) was roughly 10-fold higher than that of the common biosphere (total of 2319 ASVs, Fig. 1B and Supplementary Fig. 9). Moreover, the rare and common biospheres differ in phylum composition: the rare biosphere encompassed a larger number of bacterial phyla (total of 44) compared with the common biosphere (total of 22, Supplementary Fig. 10). Also, 22 bacterial (candidate) phyla were exclusively found within the rare biosphere. The composition of ASVs was significantly different between the rare and common biospheres (PERMANOVA *R*^*2*^ = 0.12, *P* < 0.01; Fig. 1C and Supplementary Table 2), and there was even distinct community composition across each successional stage (PERMANOVA *R*^*2*^ = 0.27, *P* < 0.01). As succession proceeds, the level of divergence between the rare and common biosphere increases progressively (Fig. 1C). In line with this observation, PERMANOVA analysis revealed a significant interaction between successional stages and the community component (rare and common biospheres; PERMANOVA *R*^*2*^ = 0.20, *P* < 0.01). It is worth mentioning that similar patterns of β-diversity were obtained using the rarity cutoff values of 0.2% and 0.05% (see Supplementary Fig. 11 and Table 2).

### Types of rarity and commonness

**Fig. 2:**
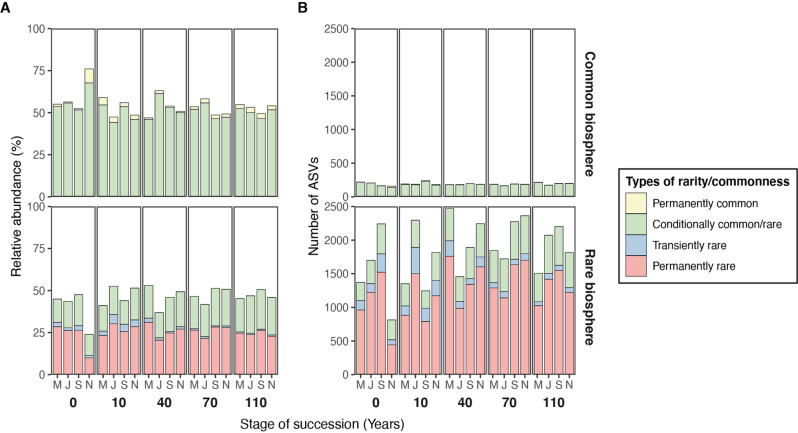
Variation in the types of rarity and commonness over space and time. The relative abundance (**A**) and number of ASVs (**B**) of each type of rarity and commonness in the rare and common biospheres across four sampling times (M-May, J-July, S-September, N-November) and five successional stages (0, 10, 40, 70 and 110 years in succession). Colors represent different types of rarity or commonness. The relative abundances and number of ASVs correspond to the average values among three replicates.

By checking the abundance change across successional stages and sampling time points, we identified a total of 11 838 permanently rare ASVs (encompassing 38 phyla), 8333 transiently rare ASVs (42 phyla), 2197 conditionally rare/common ASVs (21 phyla), and 122 permanently common ASVs (11 phyla) using the rarity cutoff of 0.1% (Supplementary Fig. 12). By quantifying the abundance and richness of each type of rarity/commonness, we found that permanently rare and conditionally rare ASVs are the dominant types of rarity in the rare biosphere (Fig. 2A, B; Supplementary Table 3). In terms of relative abundance, more than half of the rare biosphere is represented by permanently rare ASVs (54.73 ± 0.85%, average ± standard error), followed by conditionally rare ASVs (41.13 ± 0.96%). This occurs even though the number of conditionally rare ASVs (437 ± 15 ASVs) is far lower than that of permanently rare ASVs (1 258 ± 53 ASVs). Last, transiently rare ASVs (139 ± 13 ASVs) only constituted 4.14 ± 0.42% of the total abundance of the rare biosphere. On the other hand, for the common biosphere, conditionally common ASVs (185 ± 4 ASVs) formed the dominant type of commonness, which encompassed 95.84 ± 0.41% of the total abundance of the common biosphere, whereas permanently common ASVs (5 ± 1 ASVs) only encompassed 4.16 ± 0.41% of the total abundance (Fig. 2A, B; Supplementary Table 3).

Notably, the composition of each type of rarity/commonness also changed when increasing the stringency of the rarity cutoff from 0.2% to 0.05%. Regarding the rare biosphere, the total relative abundance decreased as the rarity cutoff became stricter, i.e., 61.59%, 45.65%, and 28.36% at rarity cutoff values of 0.2%, 0.1%, and 0.05%, respectively. Moreover, as the rarity cutoff became stricter, the proportion of permanently and transiently rare ASVs decreased, while conditionally rare ASVs increased (Supplementary Figs. 13, 14 and Table 3). Regarding the common biosphere, the proportion of conditionally common ASVs decreased, while permanently common increased as the rarity cutoff became stricter (Supplementary Figs. 13, 14 and Table 3).

### The influence of assembly processes structuring the rare and common biospheres

**Fig. 3:**
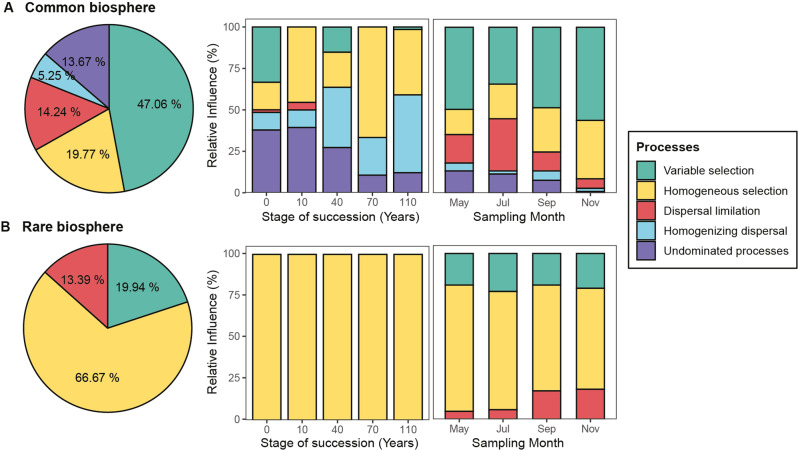
Relative influence of assembly processes structuring the soil bacterial communities. (**A**) the common biosphere and (**B**) the rare biosphere. Pie plots show the relative influence of each assembly process shapes the species turnover of the rare and common biospheres across all samples. Bar plots in the middle and right show the interplay of assembly processes governing the temporal variation of communities within each successional stage (i.e., 0, 10, 40, 70, and 110 years in succession), and the assembly processes driving the spatial variation of communities across successional stages at four sampling times (i.e., May, July, September, and November). The βNTI and RC_bray_ values were used to quantify the relative importance of each assembly process. Colors represent different assembly processes, i.e., variable selection, homogeneous selection, dispersal limitation, homogenizing dispersal, and undominated processes. The asterisk denotes the impact of homogenizing dispersal and undominated processes for the turnover of the whole community, which are 0.56% and 0.06%, respectively.

By comparing the phylogenetic and taxonomic structure of sub-communities with null expectations, we quantified the ecological processes structuring the rare and common biospheres. Our results revealed the assembly of the bacterial rare and common biospheres to be mediated by distinct ecological processes. Overall, selection influenced the assembly of the rare and common biospheres, which accounted for 86.61% and 66.83%, respectively. However, the taxa turnover of the rare biosphere was governed mainly by homogeneous selection (66.67%, Fig. 3B). Variable selection and dispersal limitation also had moderate influences on the rare biosphere, i.e., 19.94% and 13.39%, respectively. On the other hand, the taxa turnover of the common biosphere was mostly governed by variable selection (47.06%). In contrast, variable selection, dispersal limitation, undominated processes, and homogenizing dispersal were found to exert less important roles (19.77%, 14.24, 13.67, and 5.25%, respectively; Fig. 3A).

The data obtained from community assembly models were further used to investigate patterns in both temporal (within each stage of succession) and spatial (across stages of succession) variations of the rare and common biospheres. Homogeneous selection dominated the temporal assembly of the rare biosphere at all soil successional stages (100%, Fig. 3B). The relative influences of homogeneous selection were greater for the temporal rather than the spatial turnover of both the rare and common biospheres (Fig. 3A, B). In contrast, the relative influences of variable selection were greater for the spatial rather than the temporal turnover of both the rare and common biospheres (Fig. 3A, B). This result illustrates the selection exerted by the different soil conditions across different successional stages, in contrast to similar conditions within each stage. For the common biosphere, homogenizing dispersal and undominated processes had a greater influence on the temporal rather than the spatial community turnover of the common biosphere (Fig. 3A). Homogenizing dispersal was more pronounced at late successional stages and displayed a progressive increase in importance as succession proceeds, while undominated processes gradually decreased in importance along the succession (Fig. 3A). Notably, here, the ‘homogenizing dispersal’ contributing to the temporal changes at the same stage should be interpreted as the species composition was highly consistent at different months rather than migration among locations.

Similar assembly processes were identified when we used the rarity cutoff values of 0.2% and 0.05% to define the rare biosphere (Supplementary Fig. 15). Specifically, the dominant roles of homogeneous selection in structuring the rare biosphere and variable selection in structuring the common biosphere were observed at all the tested rarity cutoffs, i.e., from 0.2 to 0.05% (Supplementary Fig. 15). For both the rare and common biospheres, the relative importance of homogeneous selection gradually decreased as rarity cutoff value was reduced from 0.2 to 0.05%, whereas the extent of variable selection and dispersal limitation slightly increased (the relative influence of variable selection changed from 19.04 to 25.03%; the relative influence of dispersal changed from 11.53% to 16.05%).

## Discussion

The microbial rare biosphere is crucial for maintaining ecosystem diversity and functioning. However, it remains unknown how this significant component of microbial communities is structured and which ecological processes such as selection, dispersal, drift, and diversification contribute to the dynamics of this large proportion of low abundant microbes. Using a primary succession system, where variation in soil physicochemical characteristics coexists with similar source communities, we showed that selection was the main driver of both rare and common biospheres (86.61% and 66.83%, respectively). In our model analysis, selection is inferred when variation in the phylogenetic structure of communities significantly differs from the null expectation. Most importantly, the use of phylogenetic analysis holds the assumption of phylogenetic niche conservatism, that is, phylogenetically related species have similar ecological traits. Such an assumption can be further tested using phylogenetic signal analysis [28, 54]. This overall dominance of selection corroborates previous findings that both the rare and common biospheres are sensitive to environmental conditions as they display consistent biogeographic patterns [55, 56].

### The rare biosphere turnover is mainly governed by homogeneous selection

**Fig. 4:**
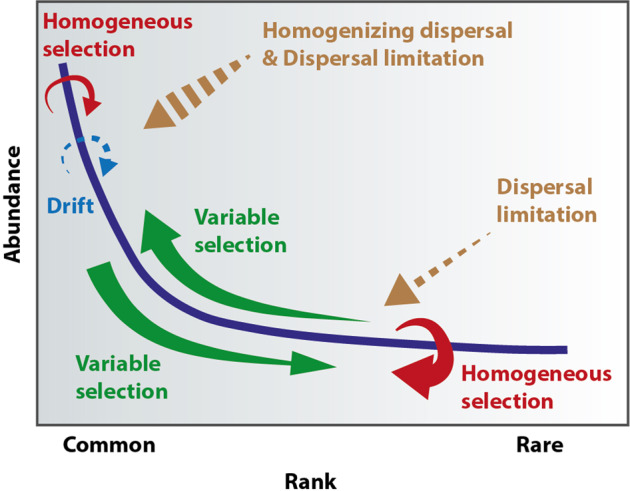
Conceptual figure displaying the relative influences of distinct ecological processes structuring the common and rare microbial biospheres. This figure depicts a rank-abundance curve in which species abundance (purple line) is ordered from high (common biosphere) to low (rare biosphere). In this study, we demonstrated that the majority of the rare species constantly remain at low abundances and are structured by homogeneous selection (red arrow). In contrast, dispersal limitation (brown arrow) plays a reduced role. Due to variable selection, a substantial fraction of the common species and the rare species shift between them across space and time (green arrows). In contrast with the rare biosphere, the abundance of common species is also driven by homogenizing dispersal (brown arrow) and drift (blue arrow), and to a lesser extent, by homogeneous selection (red arrow).

Our results reveal that an interplay of homogeneous selection, variable selection, and dispersal limitation led to higher ASV richness in the rare biosphere. Among these processes, homogeneous selection was the primary driver influencing the assembly of the rare biosphere. According to our hypothesis that homogeneous selection is related to the existence of permanently rare species, we observed this type of rarity to represent the most abundant one in the rare biosphere. This suggests that a significant fraction of the rare biosphere consisting of permanently rare taxa is not driven by biotic and abiotic variation. Instead, the environmental conditions that primarily impose selection on the rare biosphere turnover can also be constant. Similarly, a study that targeted culturable low abundant bacteria found that the relative abundance of slow-growing rare taxa was not affected by changes in nutrient concentration [57]. Together, these results suggest that permanently rare ASVs with particular sets of ecological traits (e.g., low growth rate and competitive ability) can withstand environmental fluctuation (Fig. 4, red arrow). Therefore, permanently rare taxa in our system have potential selective advantages at low abundances ensuring their long-term persistence.

Variable selection was a secondary but essential process structuring the turnover of the rare biosphere. Some soil physicochemical properties substantially changed throughout succession in this ecosystem or across sampling times, thus imposing variable selection [30]. For example, a significant change in the quality and quantity of soil organic matter has shown to be consistent with variable selection across successional stages [23]. By making the rarity cutoff stricter, we found the relative importance of variable selection increased as the relative abundance of conditionally rare ASVs increased. In line with our hypothesis, variable selection operating through changes in environmental conditions was responsible for the dynamics of conditionally rare species, which were also a dominant type of rarity in the system. This idea is supported by Kurm et al., who found that rare taxa with the ability to grow fast are affected by variation in nutrient concentration [57]. In another case, conditionally rare taxa relying on labile nitrogen increased in abundance as nitrogen limitation was alleviated [58]. These examples collectively confirm that species shifting between rare and common biospheres is related to environmental fluctuations, i.e., variable selection can be responsible for the presence of conditionally rare species (see discussion on balancing rarity and commonness and Fig. 4, green arrows).

To verify the results obtained based on Stegen’s approach, we further tested the dataset using Sloan’s neutral model [59]. However, since this model relies on species distributions, it does not work well for modeling subsets of communities [18, 60]. Thereby, we only apply this model to the whole dataset rather than using it to model the assembly of the rare biosphere. The model for the entire dataset explained 44% of the spatiotemporal variation in the metacommunity, which is higher than that inferred from the phylogenetic-based null model (<20%). By mapping rarity types on the species distribution plot, we found that distinct types of rarity and commonness show different deviations from the neutral prediction (Supplementary Fig. 16). In brief, permanently rare taxa were found more frequently than predicted by the neutral model (Supplementary Fig. 16), indicating the wide distribution of these taxa to not be restricted by dispersal. Conditionally rare taxa were often found less frequently than expected by chance (Supplementary Fig. 16), suggesting they were selected by environmental conditions and only present at conditions that meet their fitness. As such, results from the Sloan’s model based on the entire dataset supported the results from the Stegen’s model, albeit the Sloan’s neutral model cannot be directly applied to partition the rare biosphere.

Last, dispersal limitation also contributed to the turnover of the rare biosphere, albeit to a lower extent than selection. This is consistent with results from a previous study that reported that rare taxa are geographically restricted [46]. Despite the differences found, the rare biosphere was governed mostly by dispersal limitation [61]. As the increase of the proportion of transiently rare taxa was correlated with the rise of dispersal limitation in the assembly of the rare biosphere when the rarity cutoff became stricter, we suggest that dispersal limitation might be necessary for the persistence of a small fraction of the transiently rare species. These transiently rare species are introduced via limited dispersal processes but cannot adapt to the new abiotic or biotic conditions (Fig. 4, brown dashed arrow). For instance, immigrant bacteria are known to face colonization resistance by the resident microbiome. In one study, transient food-borne bacteria were found to coexist with the resident gut microbiome temporarily, but biotic competition hindered its long-term persistence within the community [62]. In the studied salt marsh ecosystem, transiently rare bacteria likely resulted from restricted dispersal processes, such as marine microbes carried by the tide, terrestrial microbes dispersed by the wind, or microbes hitchhiking on eukaryotic organisms [63, 64]. These transiently rare species have often been described in communities of macro-organisms [65]; however, the dynamics and ecological roles of this type of rare species in microbial communities remain mostly elusive. Understanding the ecological basis of their assembly and dynamics will provide the initial step toward this endeavor.

By partitioning the rare and common biospheres at three cutoff values (i.e., 0.05%, 0.1%, and 0.2%), we found that the results of community assembly are highly consistent. Notably, the relative influences of distinct ecological processes and the rarity types changed in a coordinated manner when increasing the stringency of the rarity cutoff. Specifically, the influence of homogeneous selection decreased congruously with the proportion of permanently rare ASVs in the rare biosphere, and the impact of variable selection and the proportion of conditionally rare ASVs in the rare biosphere increased in a similar fashion. The increased relative influence of dispersal limitation also aligned with the increased number of transiently rare ASVs. Taken together, these results further strengthen our hypothesis that a distinct interplay of ecological processes influences each type of rarity in a likely predictable manner.

### The common biosphere turnover is mainly governed by variable selection

To contrast the processes structuring the rare biosphere, we also investigated the assembly of its counterpart, the common biosphere. Overall, the relative influences of community assembly processes changed in structuring the common biosphere as succession proceeded—an opposing pattern to that observed for the rare biosphere. Variable selection was the most dominant process across different successional stages, and sampling time, namely selection operates through environmental heterogeneity and/or biotic interactions [23]. We found the common biosphere mainly consisted of conditionally common species. These findings indicate that species in the common biosphere are likely more sensitive to biotic and abiotic fluctuations and can retract into the rare biosphere under harsh environmental conditions. For example, members of the phylum Cyanobacteria showed higher abundance in the common biosphere at early successional stages, i.e., 0 and 10 years of succession (Supplementary Fig. 10). At these sites, the vegetation coverage is patchy, which provides appropriate conditions for oxygenic photosynthetic organisms [30]. While members of phylum Cyanobacteria mostly stay in low abundance at late successional stages as the environments do not favor them anymore (Supplementary Fig. 10). This agrees with the finding that the abundance of dominant bacterial taxa fluctuates in response to disturbance treatments [66]. Similar results were also found in other ecosystems. For instance, depending on the bioavailability of organic pollutants, members of the conditionally rare taxa can become abundant and are responsible for degrading these compounds in the freshwater ecosystem [67]. In our study system, a dominant role of variable selection in structuring the common biosphere was observed at all the tested rarity cutoffs, i.e., from 0.2% to 0.05%, with the relative importance of selection increasing as rarity cutoff value was decreased. Specifically, the increased variable and homogeneous selection align with the increased number of conditionally and permanently common ASVs, respectively (Supplementary Fig. 14). This further proved that different types of commonness are influenced by different ecological processes.

Undominated processes and homogenizing dispersal also influenced the assembly of the common but not the rare biosphere. Previous data in communities of macro-organisms suggest that rare species are more subject to ecological drift than abundant species. But in our dataset, rare taxa with a relative abundance of 0.01% or one read per community could reflect as approximately 10^5^–10^6^ individuals in 2 grams of soil. With this considerable abundance, we would say that the theories applied to macro-organisms might not apply to microorganisms. The stronger influence of undominated processes in the common biosphere at the early successional stages was supported by the previous finding in the same system, which mostly focused on the members of the common biosphere due to less sequencing depth by pyrosequencing [23]. These results suggest microbial community composition at early successional stages is more prone to be influenced by ecological drift than in late successional stages. Notice that since there are fewer species in the common biosphere, the null model results in Stegen’s approach are more biased [68]. On the other hand, we found that although homogenizing process plays a minor role, it responded to the variation of community across successional stages. By analyzing microbial biogeographical patterns, Meyer et al. showed that abundant taxa are less restricted in distribution than low abundant ones [69]. A study of rare and dominant prokaryotic lineages in hydrothermal vent systems also reported that abundant lineages of archaea displayed a more cosmopolitan distribution, while rare lineages of archaea and almost all bacterial lineages are not widely dispersed [70]. For the temporal variation of communities, homogenizing dispersal found within each stage should be interpreted as species kept alive from previous times during temporal turnover. A stronger signal of homogenizing dispersal at late successional stages is actually community compositions in different sampling time points are more similar than random processes driven by ecological drift. This is in turn supported by the finding that undominated processes were less important at late successional stages. Together, these findings support the inference that abundant taxa tend to be influenced by homogenizing dispersal and display widespread distributional patterns.

### Balancing rarity and commonness

We show that the rare biosphere corresponds to about 90% of the total bacterial diversity in these soils. Given that higher diversity helps to stabilize ecosystem processes in response to perturbation, decreases community susceptibility to invasion by non-native taxa, and increases nutrient cycling by resource partitioning [71–75], it is likely that the rare biosphere accounts for a fundamental but underestimated role in the functioning and stability of soil ecosystems. We demonstrate in this study that the ecological processes structuring the rare biosphere differ from that of the common biosphere, leading to distinct species distribution and composition. Moreover, the relative importance of different ecological processes reveals the dynamics between these components of the soil bacterial communities, as exemplified in Fig. 4. Specifically, the common biosphere consists primarily of conditionally common species, which might retract into the rare biosphere under harsh environmental conditions. Part of the rare biosphere can serve as a reservoir for these conditionally common species. Thus, they can stay at low abundances to avoid extreme conditions and respond once favorable environmental factors emerge, thus building up their population sizes (Fig. 4). When they become abundant, these conditionally rare taxa are expected to be related to the changes in the ecosystem functioning. They can also replace the previous abundant species, perform their corresponding functions and keep the resilience of ecosystems. However, the remaining part (about 2/3 of the rare biosphere) is driven mainly by homogeneous selection, restraining these species to constant lower abundances. As such, these permanently rare species are more prone to perish in response to environmental disturbances, leading to important effects on the resistance of ecosystems.

### Potential caveats and limitations

Here, we applied a quantitative approach to investigate how these processes govern the spatiotemporal turnover of the actively rare and common biospheres of bacterial communities across a natural soil chronosequence. However, this approach has intrinsic limitations for quantifying the influence of each assembly process [76–78]. First, it represents the overall influence of ecological processes at the meta-community level rather than the relative influence of these processes on microbial groups within each community [78]. Therefore, we can only quantify the relative importance of dominant processes among communities rather than within a community or a taxonomic group. Second, sequencing cluster methods also influence the inference of community assembly. For example, it is expected that ASVs will result in a greater number of sequence clusters (ASVs) and rare ASVs compared to Operational taxonomic units (OTUs). In addition, the use of ASVs can lead to lower phylogenetic and taxonomic turnover signals with higher stochastic signals when compared to OTUs [78]. We delineated ASVs from raw sequences based on the DADA2 approach rather than sequencing clustering based on 97% similarity. By controlling errors, ASVs are more sensitive to single-nucleotide variations than OTUs [37]. Therefore, we stick to the results from ASVs, which reflect actual microbes better than OTUs. Third, dispersal processes might also be overestimated when the phylogenetic turnover cannot reflect the selection. Hence, dispersal limitation can play a less critical role in the successional chronosequence (<10 km) than on large spatial scales. In addition, dispersal limitation or homogenizing dispersal cannot be the process responsible for the temporal turnover of communities at the same location when the model shows taxonomic differences. Last, ecological drift can also be inflated when the number of species is small in a community or sub-community [68]. Despite these limitations, our approach provides the first attempt to distinguish the different ecological processes structuring various types of rarity. Further experiments and data are needed to verify the findings from this study.

## Supplementary Information

Supplementary material

Supplementary material

## Data Availability

The 16S rRNA amplicon data analyzed during the current study are available in the Sequence Read Archive of the National Center for Biotechnology Information with the accession numbers PRJNA546612. All R codes used in this study are available on GitHub (https://github.com/Jia-Xiu/Jia_et_al_ISMECOMMS_2022).

## References

[1] Pedros-Alio C (2012). The rare bacterial biosphere. Ann Rev Mar Sci..

[2] Sogin ML, Morrison HG, Huber JA, Welch DM, Huse SM, Neal PR (2006). Microbial diversity in the deep sea and the underexplored “rare biosphere”. Proc Natl Acad Sci.

[3] Hausmann B, Pelikan C, Rattei T, Loy A, Pester M (2019). Long-term transcriptional activity at zero growth of a cosmopolitan rare biosphere member. mBio.

[4] Pester M, Bittner N, Deevong P, Wagner M, Loy AA (2010). ‘Rare biosphere’microorganism contributes to sulfate reduction in a peatland. ISME J.

[5] Rivett DW, Bell T (2018). Abundance determines the functional role of bacterial phylotypes in complex communities. Nat Microbiol.

[6] van Elsas JD, Chiurazzi M, Mallon CA, Elhottova D, Kristufek V, Salles JF (2012). Microbial diversity determines the invasion of soil by a bacterial pathogen. Proc Natl Acad Sci USA.

[7] Magurran AE, Henderson PA (2003). Explaining the excess of rare species in natural species abundance distributions. Nature..

[8] Rabinowitz D, Rapp JK, Dixon PM (1984). Competitive abilities of sparse grass species: means of persistence or cause of abundance. Ecology.

[9] Reinhardt K, Köhler G, Maas S, Detzel P (2005). Low dispersal ability and habitat specificity promote extinctions in rare but not in widespread species: the Orthoptera of Germany. Ecography.

[10] Yenni G, Adler PB, Ernest S (2012). Strong self-limitation promotes the persistence of rare species. Ecology..

[11] Jousset A, Bienhold C, Chatzinotas A, Gallien L, Gobet A, Kurm V (2017). Where less may be more: how the rare biosphere pulls ecosystems strings. The ISME J.

[12] Thingstad TF (2000). Elements of a theory for the mechanisms controlling abundance, diversity, and biogeochemical role of lytic bacterial viruses in aquatic systems. Limnol Oceanogr..

[13] Szekely AJ, Langenheder S (2014). The importance of species sorting differs between habitat generalists and specialists in bacterial communities. FEMS Microbiol Ecol.

[14] Mo Y, Zhang W, Yang J, Lin Y, Yu Z, Lin S (2018). Biogeographic patterns of abundant and rare bacterioplankton in three subtropical bays resulting from selective and neutral processes. ISME J.

[15] Nemergut DR, Schmidt SK, Fukami T, O’Neill SP, Bilinski TM, Stanish LF (2013). Patterns and processes of microbial community assembly. Microbiol Mol Biology Rev.

[16] Vellend M (2010). Conceptual synthesis in community ecology. Q Rev Biol.

[17] Vellend M The Theory of Ecological Communities. Princeton University Pres. 2016:61-7.

[18] Jia X, Dini-Andreote F, Falcao Salles J (2018). Community assembly processes of the microbial rare biosphere. Trends Microbiol.

[19] Stegen JC, Lin X, Fredrickson JK, Chen X, Kennedy DW, Murray CJ (2013). Quantifying community assembly processes and identifying features that impose them. ISME J.

[20] Stegen JC, Lin X, Fredrickson JK, Konopka AE. Estimating and mapping ecological processes influencing microbial community assembly. Front Microbiol. 2015;6:10.3389/fmicb.2015.00370. 10.3389/fmicb.2015.00370 PMC4416444 25983725

[21] Webb CO, Ackerly DD, McPeek MA, Donoghue MJ (2002). Phylogenies and community ecology. Ann Rev Ecol Syst.

[22] Lynch MDJ, Neufeld JD (2015). Ecology and exploration of the rare biosphere. Nat Rev Micro..

[23] Dini-Andreote F, Stegen JC, van Elsas JD, Salles JF (2015). Disentangling mechanisms that mediate the balance between stochastic and deterministic processes in microbial succession. Proc Natl Acad Sci USA.

[24] Chase JM, Kraft NJB, Smith KG, Vellend M, Inouye BD (2011). Using null models to disentangle variation in community dissimilarity from variation in α-diversity. Ecosphere.

[25] Shade A, Jones SE, Caporaso JG, Handelsman J, Knight R, Fierer N (2014). Conditionally rare taxa disproportionately contribute to temporal changes in microbial diversity. mBio.

[26] Strous M, Heijnen JJ, Kuenen JG, Jetten MSM (1998). The sequencing batch reactor as a powerful tool for the study of slowly growing anaerobic ammonium-oxidizing microorganisms. Appl Microbiol Biotechnol.

[27] Goldfarb KC, Karaoz U, Hanson CA, Santee CA, Bradford MA, Treseder KK (2011). Differential growth responses of soil bacterial taxa to carbon substrates of varying chemical recalcitrance. Front Microbiol.

[28] Jia X, Dini-Andreote F, Falcao Salles J. Comparing the influence of assembly processes governing bacterial community succession based on DNA and RNA Data. Microorganisms. 2020;8. 10.3390/microorganisms8060798. 10.3390/microorganisms8060798 PMC7355735 32466517

[29] Olff H, De Leeuw J, Bakker JP, Platerink RJ, van Wijnen HJ (1997). Vegetation succession and herbivory in a salt marsh: changes induced by sea level rise and silt deposition along an elevational gradient. J Ecol.

[30] Dini-Andreote F, Silva M, Triado-Margarit X, Casamayor EO, van Elsas JD, Salles JF (2014). Dynamics of bacterial community succession in a salt marsh chronosequence: evidences for temporal niche partitioning. ISME J.

[31] Dini-Andreote F, Pylro VS, Baldrian P, van Elsas JD, Salles JF (2016). Ecological succession reveals potential signatures of marine–terrestrial transition in salt marsh fungal communities. ISME J.

[32] Schrama M, Berg MP, Olff H (2012). Ecosystem assembly rules: the interplay of green and brown webs during salt marsh succession. Ecology..

[33] Caporaso JG, Lauber CL, Walters WA, Berg-Lyons D, Lozupone CA, Turnbaugh PJ (2011). Global patterns of 16S rRNA diversity at a depth of millions of sequences per sample. Proc Natl Acad Sci.

[34] Caporaso JG, Lauber CL, Walters WA, Berg-Lyons D, Huntley J, Fierer N (2012). Ultra-high-throughput microbial community analysis on the Illumina HiSeq and MiSeq platforms. ISME J.

[35] Carini P, Marsden PJ, Leff JW, Morgan EE, Strickland MS, Fierer N (2016). Relic DNA is abundant in soil and obscures estimates of soil microbial diversity. Nat Microbiol.

[36] Bolyen E, Rideout JR, Dillon MR, Bokulich NA, Abnet C, Al-Ghalith GA (2018). QIIME 2: Reproducible, interactive, scalable, and extensible microbiome data science. PeerJ Preprints.

[37] Callahan BJ, McMurdie PJ, Holmes SP (2017). Exact sequence variants should replace operational taxonomic units in marker-gene data analysis. ISME J.

[38] Yilmaz P, Parfrey LW, Yarza P, Gerken J, Pruesse E, Quast C (2014). The SILVA and “All-species Living Tree Project (LTP)” taxonomic frameworks. Nucleic Acids Res.

[39] Price MN, Dehal PS, Arkin AP (2009). FastTree: computing large minimum evolution trees with profiles instead of a distance matrix. Mol Biol Evol.

[40] R Core Team: R: A language and environment for statistical computing. In. Vienna, Austria: R Foundation for Statistical Computing; 2017.

[41] RStudio Team: RStudio: integrated development for R. In., vol. 42. Boston, MA: RStudio, Inc.; 2015.

[42] Wickham H (2010). ggplot2: elegant graphics for data analysis. J Stat Softw.

[43] Chen H, Boutros PC (2011). VennDiagram: a package for the generation of highly-customizable Venn and Euler diagrams in R. BMC Bioinf.

[44] Dixon P (2003). VEGAN, a package of R functions for community ecology. J Veg Sci.

[45] Yamamoto K, Hackley KC, Kelly WR, Panno SV, Sekiguchi Y, Sanford RA, et al. Diversity and geochemical community assembly processes of the living rare biosphere in a sand-and-gravel aquifer ecosystem in the Midwestern United States. Sci Rep. 2019;9. 10.1038/s41598-019-49996-z. 10.1038/s41598-019-49996-z PMC6748922 31530884

[46] Galand PE, Casamayor EO, Kirchman DL, Lovejoy C (2009). Ecology of the rare microbial biosphere of the Arctic Ocean. Proc Natl Acad Sci.

[47] Reveillaud J, Maignien L, Murat Eren A, Huber JA, Apprill A, Sogin ML (2014). Host-specificity among abundant and rare taxa in the sponge microbiome. ISME J.

[48] Logares R, Audic S, Bass D, Bittner L, Boutte C, Christen R (2014). Patterns of rare and abundant marine microbial eukaryotes. Curr Biol.

[49] Campbell BJ, Yu L, Heidelberg JF, Kirchman DL (2011). Activity of abundant and rare bacteria in a coastal ocean. Proc Natl Acad Sci.

[50] Hirsch JE (2005). An index to quantify an individual’s scientific research output. Proc Natl Acad Sci USA.

[51] Haegeman B, Hamelin J, Moriarty J, Neal P, Dushoff J, Weitz JS (2013). Robust estimation of microbial diversity in theory and in practice. ISME J.

[52] Paradis E, Claude J, Strimmer K (2004). APE: analyses of phylogenetics and evolution in R language. Bioinformatics (Oxford, England).

[53] Kembel SW, Cowan PD, Helmus MR, Cornwell WK, Morlon H, Ackerly DD (2010). Picante: R tools for integrating phylogenies and ecology. Bioinformatics..

[54] Stegen JC, Lin X, Konopka AE, Fredrickson JK (2012). Stochastic and deterministic assembly processes in subsurface microbial communities. ISME J.

[55] Jiao S, Lu Y (2020). Soil pH and temperature regulate assembly processes of abundant and rare bacterial communities in agricultural ecosystems. Environ Microbiol.

[56] Logares R, Lindström ES, Langenheder S, Logue JB, Paterson H, Laybourn-Parry J (2012). Biogeography of bacterial communities exposed to progressive long-term environmental change. ISME J.

[57] Kurm V, van der Putten WH, Weidner S, Geisen S, Snoek BL, Bakx T (2019). Competition and predation as possible causes of bacterial rarity. Environ Microbiol.

[58] Aanderud ZT, Saurey S, Ball BA, Wall DH, Barrett JE, Muscarella ME (2018). Stoichiometric shifts in Soil C:N:P promote bacterial taxa dominance, maintain biodiversity, and deconstruct community assemblages. Front Microbiol.

[59] Sloan WT, Woodcock S, Lunn M, Head IM, Curtis TP (2007). Modeling taxa-abundance distributions in microbial communities using environmental sequence data. Microb Ecol.

[60] Magurran AE, McGill BJ. Biological diversity: frontiers in measurement and assessment. Oxford University Press; 2011.

[61] Richter-Heitmann T, Hofner B, Krah FS, Sikorski J, Wust PK, Bunk B (2020). Stochastic dispersal rather than deterministic selection explains the spatio-temporal distribution of soil bacteria in a temperate grassland. Front Microbiol.

[62] Ivanov II, Honda K (2012). Intestinal commensal microbes as immune modulators. Cell Host Microbe.

[63] van Veelen HPJ, Falcao Salles J, Tieleman BI (2017). Multi-level comparisons of cloacal, skin, feather and nest-associated microbiota suggest considerable influence of horizontal acquisition on the microbiota assembly of sympatric woodlarks and skylarks. Microbiome.

[64] Warmink JA, Nazir R, Corten B, van Elsas JD (2011). Hitchhikers on the fungal highway: The helper effect for bacterial migration via fungal hyphae. Soil Biology Biochem.

[65] Snell Taylor SJ, Evans BS, White EP, Hurlbert AH (2018). The prevalence and impact of transient species in ecological communities. Ecology.

[66] Kurm V, Geisen S, Gera Hol WH (2019). A low proportion of rare bacterial taxa responds to abiotic changes compared with dominant taxa. Environ Microbiol.

[67] Wang Y, Hatt JK, Tsementzi D, Rodriguez RL, Ruiz-Perez CA, Weigand MR (2017). Quantifying the Importance of the Rare Biosphere for Microbial Community Response to Organic Pollutants in a Freshwater Ecosystem. Appl Environ Microbiol.

[68] Cao J, Jia X, Pang S, Hu Y, Li Y, Wang Q. Functional structure, taxonomic composition and the dominant assembly processes of soil prokaryotic community along an altitudinal gradient. Appl Soil Ecol. 2020;155. 10.1016/j.apsoil.2020.103647.

[69] Meyer KM, Memiaghe H, Korte L, Kenfack D, Alonso A, Bohannan BJM (2018). Why do microbes exhibit weak biogeographic patterns. ISME J.

[70] Anderson RE, Sogin ML, Baross JA (2015). Biogeography and ecology of the rare and abundant microbial lineages in deep-sea hydrothermal vents. FEMS Microbiol Ecol.

[71] Mallon CA, Le Roux X, van Doorn GS, Dini-Andreote F, Poly F, Salles JF (2018). The impact of failure: unsuccessful bacterial invasions steer the soil microbial community away from the invader’s niche. ISME J.

[72] Langenheder S, Bulling MT, Solan M, Prosser JI (2010). Bacterial biodiversity-ecosystem functioning relations are modified by environmental complexity. PLoS One.

[73] Bardgett RD, Van Der Putten WH (2014). Belowground biodiversity and ecosystem functioning. Nature..

[74] Griffiths B, Ritz K, Wheatley R, Kuan H, Boag B, Christensen S (2001). An examination of the biodiversity–ecosystem function relationship in arable soil microbial communities. Soil Biol Biochem.

[75] Hooper DU, Chapin F, Ewel J, Hector A, Inchausti P, Lavorel S (2005). Effects of biodiversity on ecosystem functioning: a consensus of current knowledge. Ecol Monogr.

[76] Logares R, Tesson SVM, Canback B, Pontarp M, Hedlund K, Rengefors K (2018). Contrasting prevalence of selection and drift in the community structuring of bacteria and microbial eukaryotes. Environ Microbiol.

[77] Zhou J, Ning D (2017). Stochastic community assembly: does it matter in microbial ecology?. Microbiol Mol Biol Rev.

[78] Logares R, Deutschmann IM, Junger PC, Giner CR, Krabberod AK, Schmidt TSB (2020). Disentangling the mechanisms shaping the surface ocean microbiota. Microbiome.

[79] Dini-Andreote F, Brossi MJ, van Elsas JD, Salles JF (2016). Reconstructing the genetic potential of the microbially-mediated nitrogen cycle in a salt marsh ecosystem. Front Microbiol.

