# Supplementary material for: Unravelling the interplay of ecological processes structuring the bacterial rare biosphere
Source: ISME Commun. 2022 Oct 3;2:96. doi: 10.1038/s43705-022-00177-6 (PMC9723687; doi:10.1038/s43705-022-00177-6)
Supplement: Supplementary file 2 — Supplementary material [file 43705_2022_177_MOESM2_ESM.pdf]

Supplementary material

Unravelling the interplay of ecological processes shaping the bacterial rare biosphere

Xiu Jia, Francisco Dini-Andreote, Joana Falcão Salles

Methods

Soil sampling

Soil samples were collected across five successional stages (i.e. 0, 10, 40, 70 and 110 years of development from 1809 to 2017) of a well-characterized soil chronosequence located on the island of Schiermonnikoog, the Netherlands (53°30’ N, 6°10’ E) in May, July, September and November 2017 [1]. Similar sampling sites and times were used in previous studies [2, 3]. At each successional stage, we established three replicate plots (5 × 5 m). At each plot, we randomly sampled 20 soil cores (top 10 cm), which were homogenized and used as one pooled sample per plot. A total of 2 g of each homogenized soil sample per plot was directly preserved in LifeGuard Soil Preservation Solution (Qiagen, Germany) for further RNA extraction. Preserved soil samples were stored at -80°C prior to RNA extraction.

RNA isolation, cDNA synthesis and bacterial 16S rRNA sequencing

To capture the putatively ‘active’ bacterial (i.e. excluding relic DNA) from soil, soil RNA extractions were carried out using the RNeasy PowerSoil Total RNA kit (Qiagen, Germany), following the manufacturer’s instructions. DNA was digested from RNA samples using the DNase Max kit (Qiagen, Germany). The DNA-free RNA was reverse transcribed into cDNA using the Transcriptor High Fidelity cDNA Synthesis Kit (Roche, Switzerland). The cDNA samples were then purified using the MinElute PCR Purification Kit (Qiagen, Germany). The concentration of cDNA was quantified using NanoDrop 2000 Spectrophotometer (Thermo Scientific, USA).

Bacterial community profiling was carried out by sequencing the 16S rRNA from the cDNA samples. The V4 region of bacterial 16S rRNA was amplified using the primer set 515F (5’-GTGCCAGCMGCCGCGGTAA-3’) and 806R (5’-GGACTACHVGGGTWTCTA-AT-3’), in accordance with the Earth Microbiome Project [4, 5]. For this, each sample was given a 12-base barcode sequence that linked on the forward primer. PCR assays were performed in 25 µL of PCR with 1 µL of template DNA, 1 µL of each primer (final concentration 200 pM), 9.5 µL of MOBIO PCR water and 12.5 µL of QuantaBio’s AccuStart II PCR ToughMix (final concentration 1×). PCR started with 3 minutes at 94 °C followed by 23 cycles at 94 °C for 45 s, 50 °C for 60 s, and 72 °C for 90 s, with a final extension at 72 °C for 10 min. PCR products were quantified using PicoGreen (Invitrogen, USA) and pooled in a tube using equimolar concentrations of each sample. The sample pool was purified using AMPure XP Beads (Beckman Coulter, USA), and quantified by a Qubit fluorometer (Invitrogen, USA). Pooled amplicons were diluted to 2 nM, denatured, and then diluted to a final concentration of 6.75 pM with a 10% PhiX spike for increasing the diversity of our library. Sequencing was performed on a 151bp × 12bp × 151bp Illumina MiSeq run (Illumina, USA) at the Environmental Sample Preparation and Sequencing Facility (ESPSF) at Argonne National Laboratory using the Version 2 chemistry sequencing reagent kit [4]. All 16S rRNA sequence data analyzed in this study were deposited at the Sequence Read Archive of the National Center for Biotechnology information with the accession numbers PRJNA546612 (http://www.ncbi.nlm.nih.gov/Traces/sra)[1].

Supplementary Figures

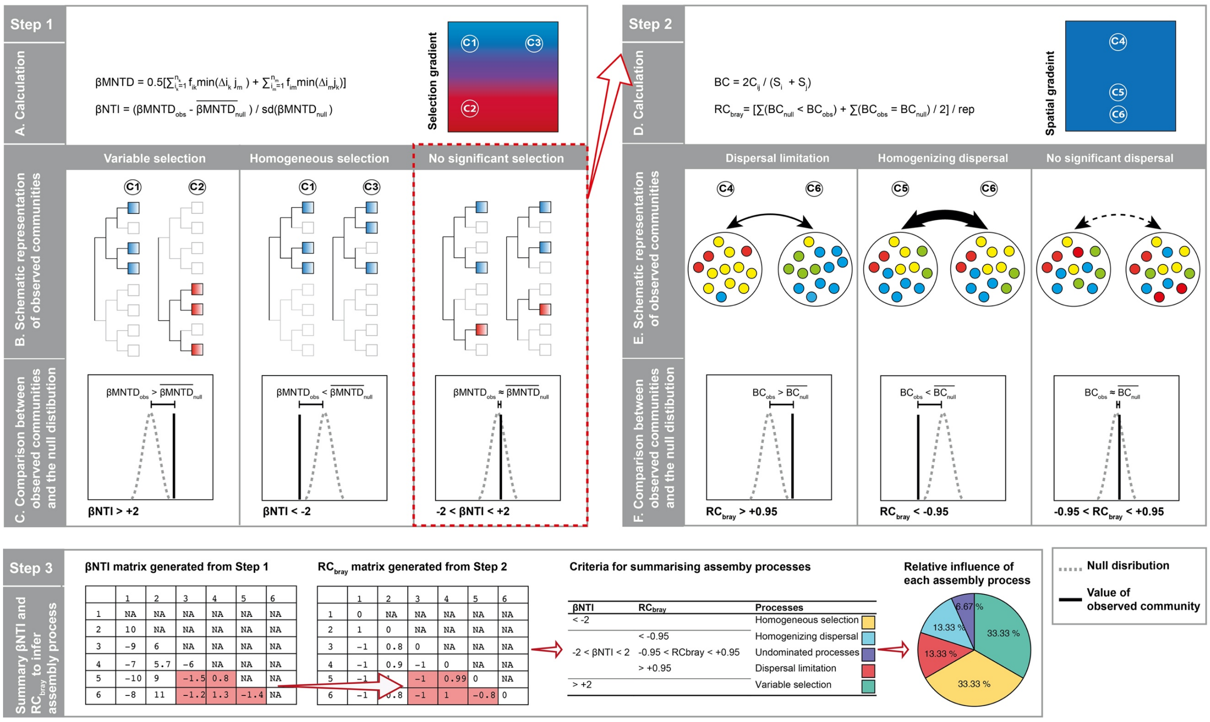

Figure 1 Methodological framework to calculate the relative influences of distinct assembly processes, using phylogenetic (Step 1) and taxonomic (Step 2) distribution (modified from Stegen *et al.*[6, 7]). In Step1 (upper left panel), **(A)** selection is inferred by the deviation of $\beta MNTD_{obs}$ from $\beta MNTD_{null}$ (i.e. βNTI value). **(B, C)** $\beta MNTD_{obs}$ (solid black lines) represents the phylogenetic distance between a given pair of communities, whereas $\beta MNTD_{null}$ (dashed grey lines) indicates the null distribution of phylogenetic turnover, generated by shuffling species in the tips of the phylogenetic tree. The predominance of variable selection leads to distinct phylogenetic species composition between two communities, e.g. communities C1 and C2 that dwell in distinct environmental conditions (illustrated in the upper right corner). In this case, the phylogenetic distance of observed communities ($\beta MNTD_{obs}$) is higher than that of the null distribution ($\beta MNTD_{null}$), i.e. βNTI > +2. Homogeneous selection (e.g. communities C1 and C3) generates a similar phylogenetic structure between observed communities in comparison with the null expectation, i.e. βNTI < -2. Non-significant deviation of βMNTD between observed communities and the null distribution indicates the absence of selection, i.e. that dispersal and/or drift processes govern community turnover (-2 < βNTI < +2). In Step2 (upper right panel), **(D)** dispersal and /or drift are further quantified by the Bray-Curtis (BC) based Raup-Crick (RC_{bray}). **(E, F)** This is done by calculating the deviation in the taxonomic difference (Bray-Curtis) between a given pair of observed communities (BC_{obs}, solid black lines) and a randomly sampled distribution (BC_{null}, dashed grey lines). Dispersal limitation leads to a significant distinct community composition between a given pair of communities (e.g. communities C4 and C6), i.e. $RC_{bray} > +0.95$. On the contrary, the predominance of homogenizing dispersal generates a significant clustering between a given pair of communities (e.g. communities C5 and C6), i.e. $RC_{bray} < -0.95$. When neither selection nor dispersal is significant, i.e. neither βNTI nor RC_{bray} are significantly different from the null distribution, the combination of drift, dispersal and selection (termed as undominated processes) is responsible for the random pattern in community turnover. In Step 3 (lower panel), the βNTI and RC_{bray} matrices acquired from Step 1 and Step 2, are used to calculate the fraction of pairwise comparisons with significant values, which infer the relative influences of distinct assembly processes. In steps 1 and 2, circles with numbers indicate communities located in different locations and/or environmental gradients.

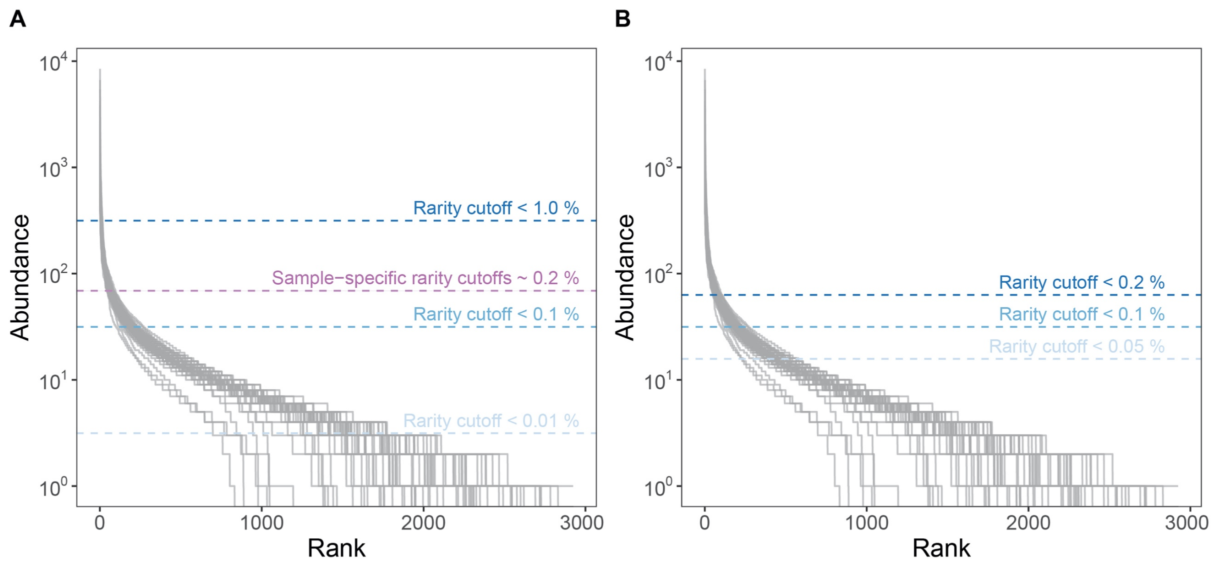

Figure 2 Rarity cutoff values based on the rank abundance curves of all samples (grey lines). The *x*-axis displays the abundance of amplicon sequence variants (ASVs) on a log scale, and the *y*-axis displays the rank of their abundances. The rarity cutoff values are shown as dashed lines and their respective percentages are indicated in the panels. (A) Rarity cutoff values are commonly used in the literature (1.0%, 0.1% and 0.01%; blue lines, e.g. ref. [8-11]) and their fit on our dataset. The orange line indicates the average of sample-specific cutoff, i.e. 0.2% (for further detail, see Figure 5). (B) Distinct rarity cutoff values were tested in this study (0.2%, 0.1% and 0.05%; blue lines).

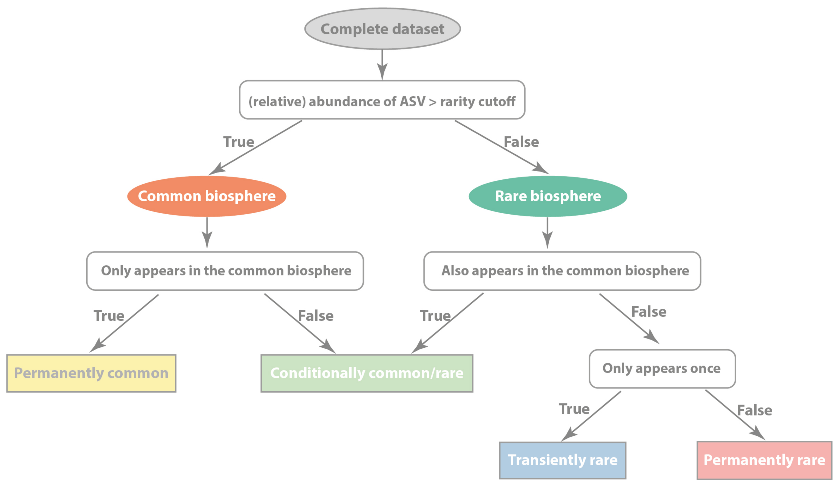

Figure 3 Workflow for defining the common and rare biospheres and classifying the different types of rarity and commonness, i.e. permanently common, conditionally rare/common, transiently rare and permanently rare.

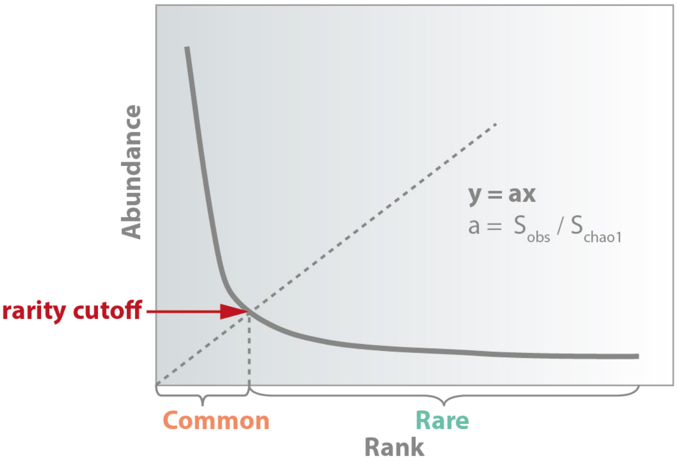

Figure 4 Conceptual figure displaying the method used to define the sample-specific rarity cutoff based on the rank abundance curve of a community (green line). Species with abundances above the intercept line ($y = ax$, orange line) are classified as common, and those below as rare. The slope of the intercept line (a) represents the sequencing depth, i.e. the ratio of the value of observed species (S_{obs}) to the value of the estimated species (S_{chao1}).

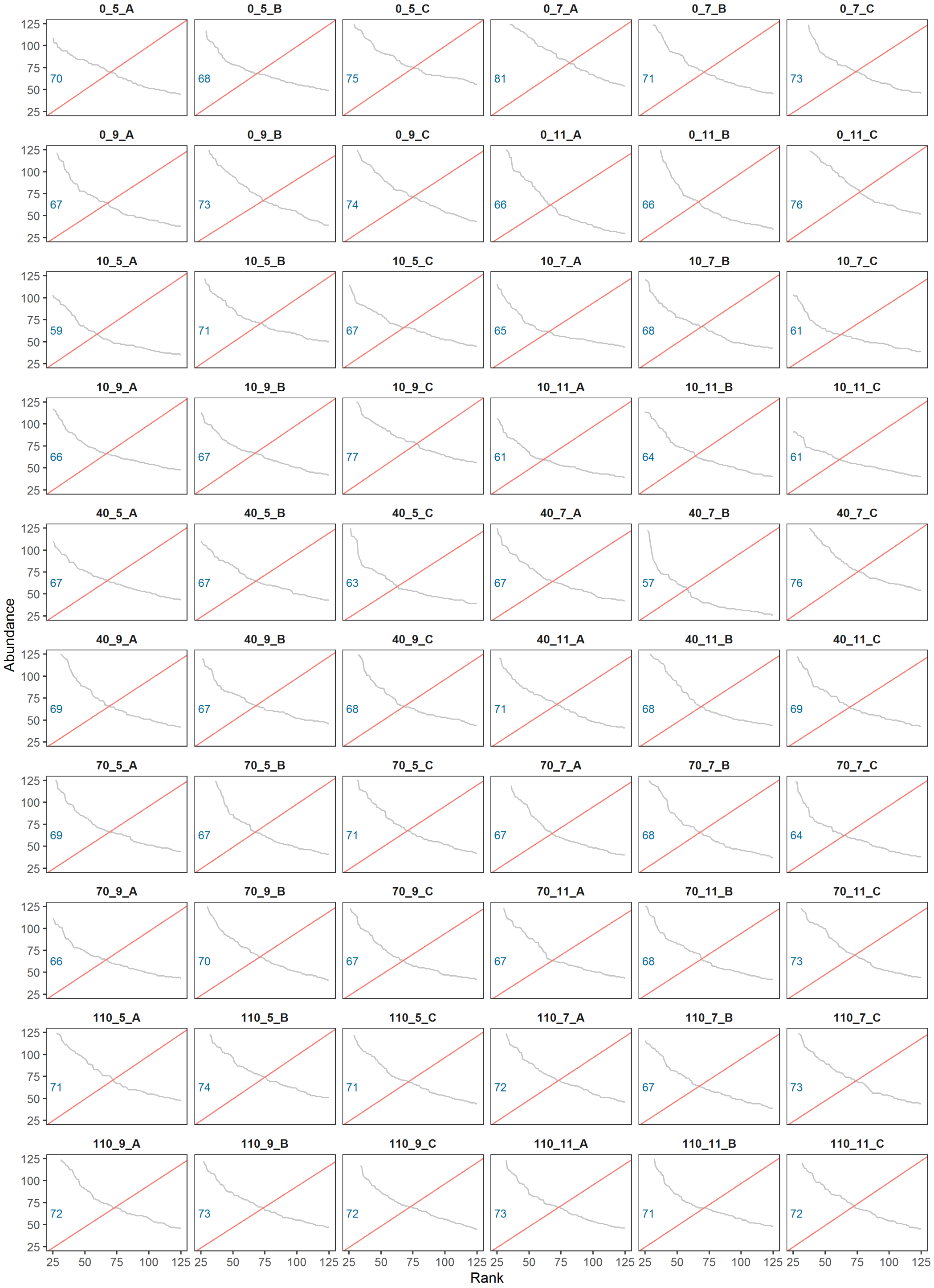

Figure 5 Panels displaying the sample-specific rarity cutoffs of each individual sample in our dataset. The sample-specific rarity cutoff values are set between 57 and 81 reads (numbers in blue). ASVs with read counts below these values are defined as rare, and those above as common. The average of the sample-specific rarity cutoffs is 69 reads, which equals to 0.2% of the total abundance per sample. This value is based on a rarified ASV table at 31,500 reads per sample. Partial of the rank abundance curve in each sample is shown by grey lines. Red lines indicate the recalibrated intercept used to identify the sample-specific rarity cutoffs. Sample IDs on top of the panels indicate the successional stage, sampling month, and replicate (separated by underscores).

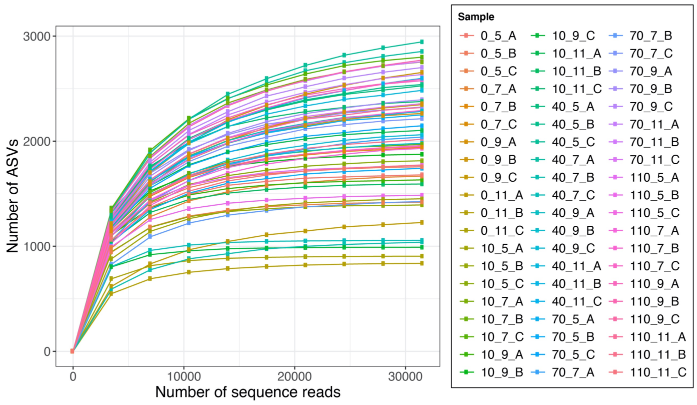

Figure 6 Rarefaction curves of individual samples. Curves are visualized by the observed number of amplicon sequence variance (ASVs) against the number of sequence reads. Lines with different colors indicate different samples/communities. Sample IDs in the legend indicate the successional stage, sampling month, and replicate (separated by underscores).

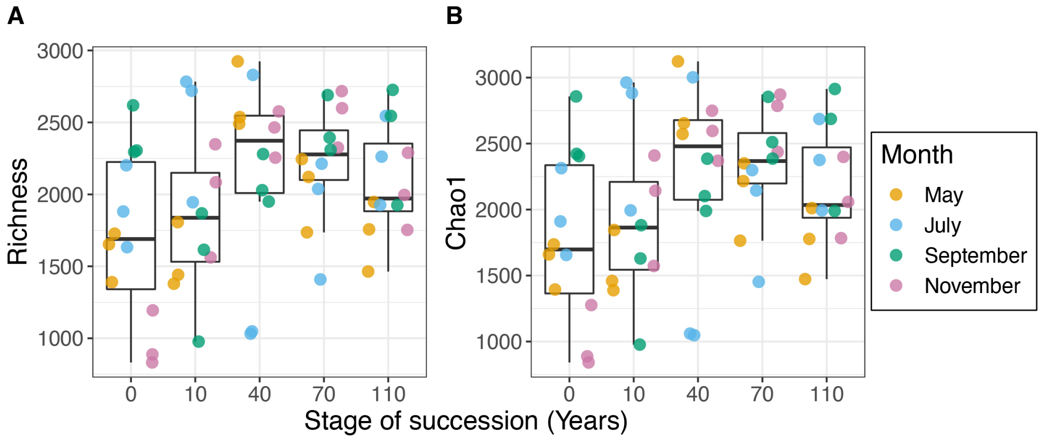

Figure 7 Box plots displaying α-diversity indices (i.e. richness, Chao1). Median values and interquartile ranges are indicated in the plots. The panels display values across successional stages (i.e. 0, 10, 40, 70 and 110 years) and sampling time (i.e. May, July, September and November).

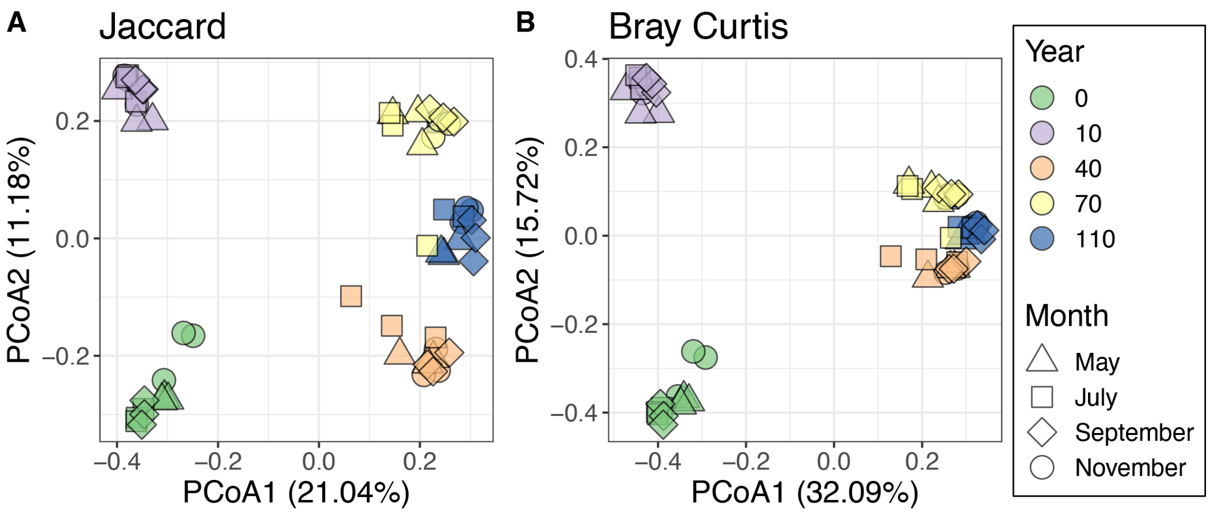

Figure 8 Principal Coordinate Analyses (PCoA) displaying bacterial community β-diversities across successional stages (i.e. 0, 10, 40, 70 and 110 years) and sampling time (i.e. May, July, September and November). (A) PCoA plot based on Jaccard distances. (B) PCoA plot based on Bray-Curtis distances.

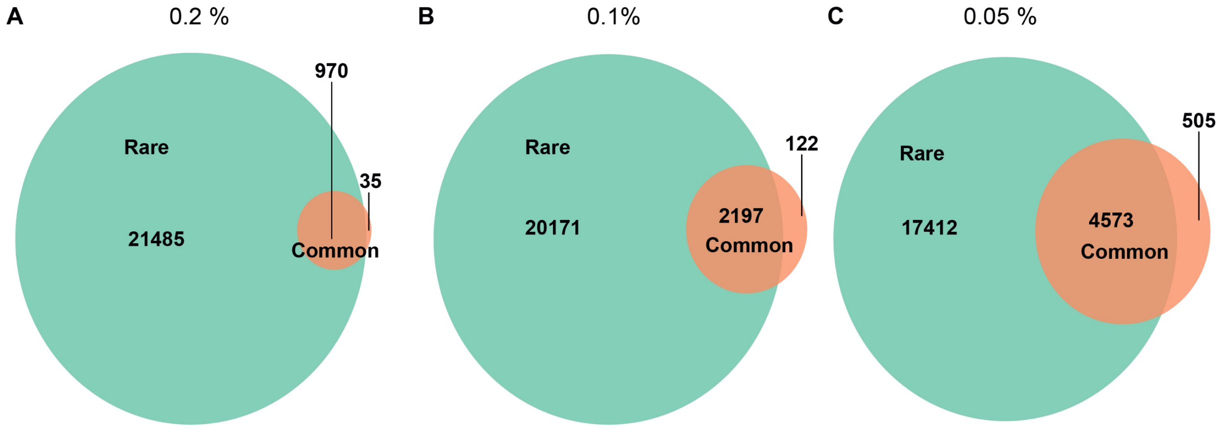

Figure 9 Venn diagrams indicating the number of amplicon sequence variants (ASVs) in the rare (green circle) and common (orange circle) biospheres. Values are shown at rarity cutoffs of (A) 0.2%, (B) 0.1% and (C) 0.05%.

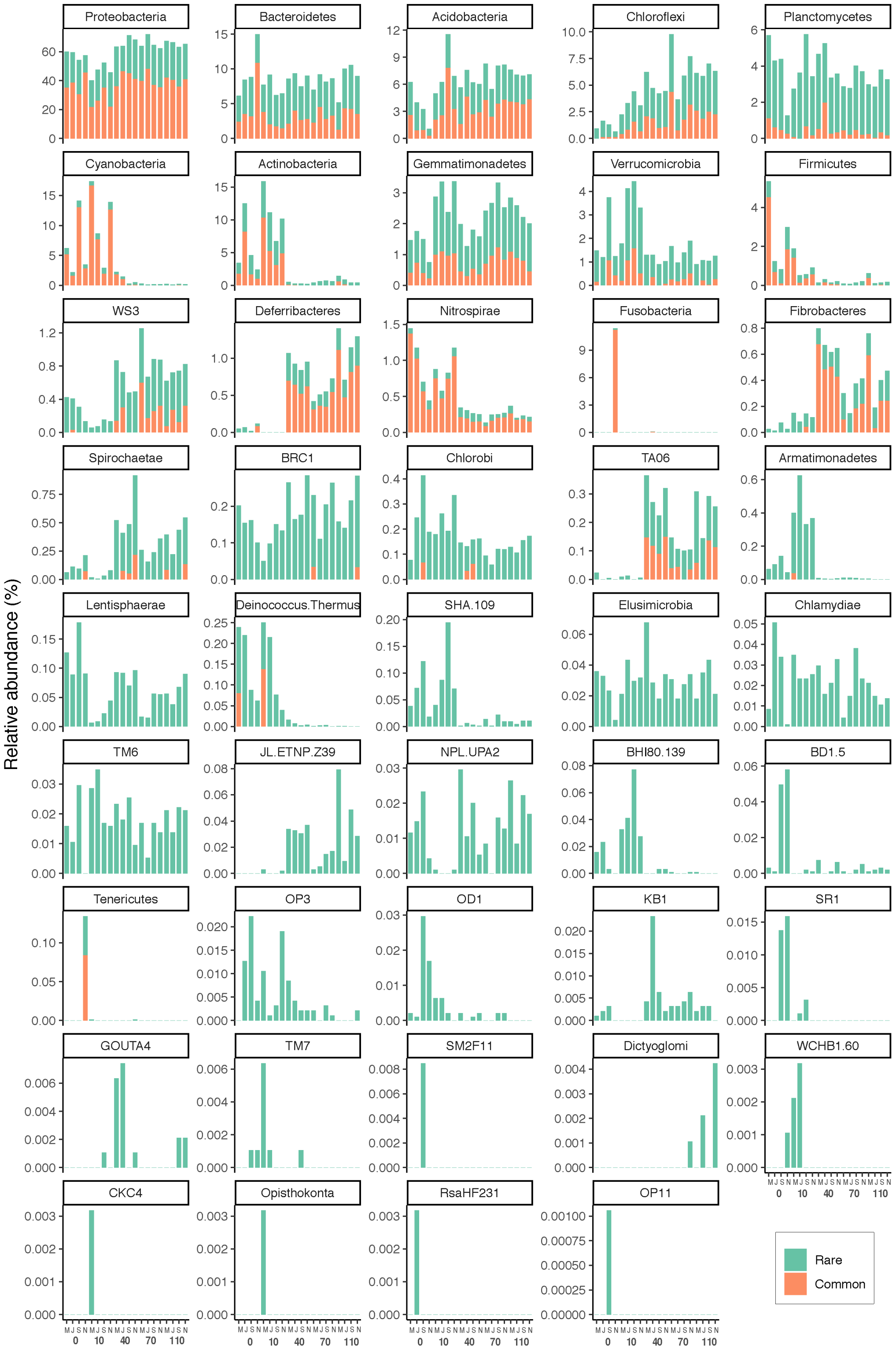

Figure 10 Bar charts displaying the relative abundances of rare and common species per bacterial phyla (green and orange bars, respectively). The rare and common biospheres were defined by the rarity cutoff of 0.1%. The height of each bar represents the average relative abundance of each phylum in the corresponding sampling group. The x-axis displays the sampling time (M-May, J-July, S-September and N-November) and successional stage (0, 10, 40, 70 and 110 years), and the y-axis displays the relative abundances (in %).

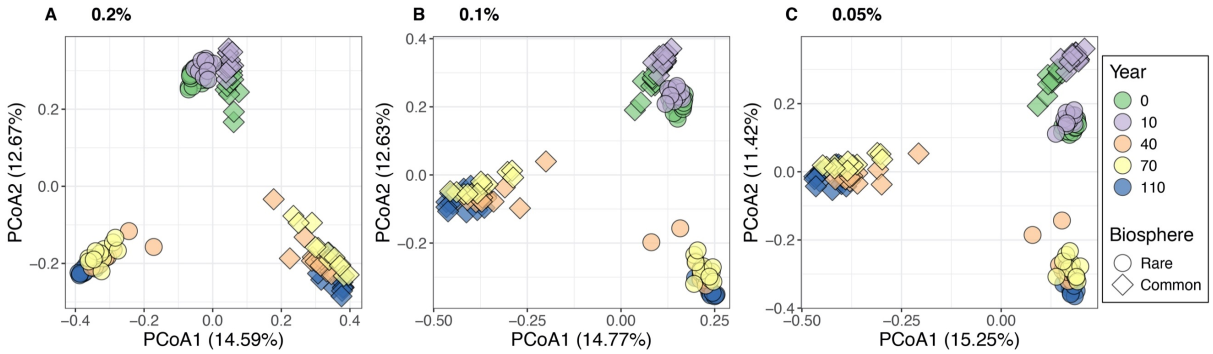

Figure 11 Principal Coordinate analyses (PCoA) based on Bray-Curtis distances of bacterial communities across successional stages (i.e. 0, 10, 40, 70 and 110 years), separated by the rare and common biospheres. Each plot displays the result at a different cutoff value: (A) 0.2%, (B) 0.1% and (C) 0.05%.

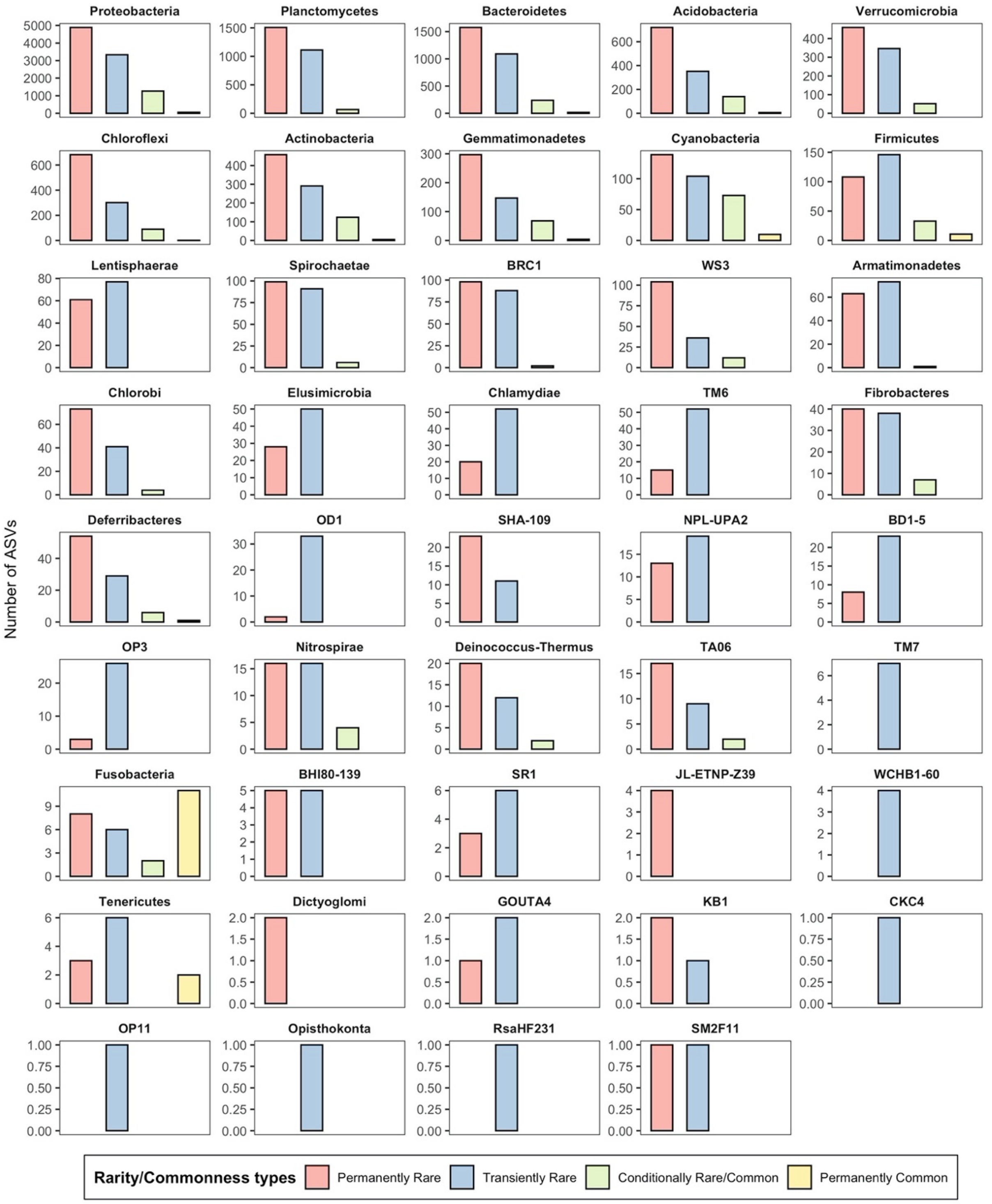

Figure 12 Bar charts displaying the number of amplicon sequence variants (ASVs) in each type of rarity and commonness per bacterial phylum. The rare and common biospheres were defined at the rarity cutoff of 0.1%. The height of each bar represents the number of ASVs.

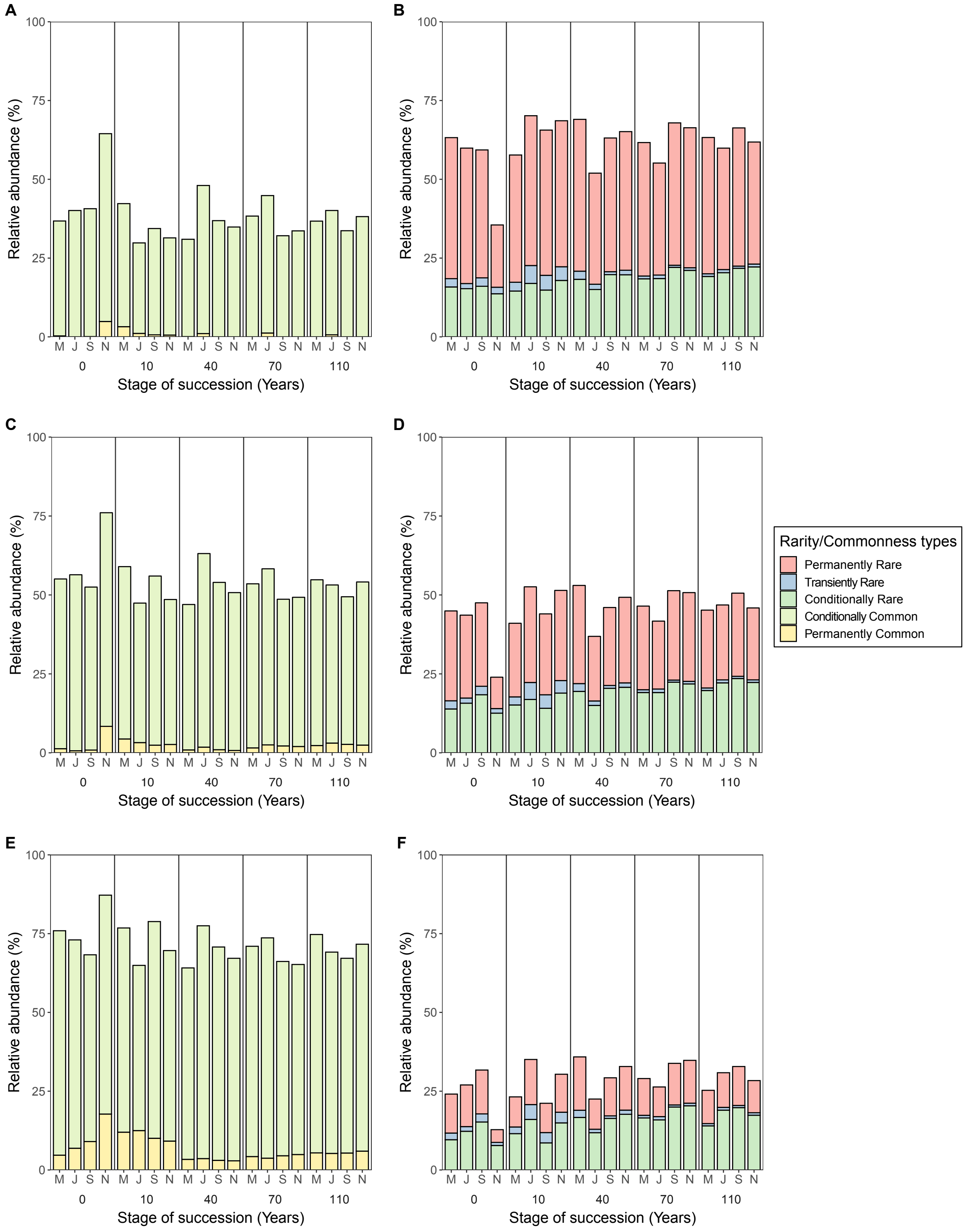

Figure 13 Bar charts displaying the relative abundance of each type of commonness (A, C and E) and rarity (B, D and F). The plots display changes across five successional stages (0, 10, 40, 70 and 110 years) and four sampling times (M-May, J-July, S-September, N-November). The common and rare biospheres are shown at distinct rarity cutoffs: (A, B) 0.2%, (C, D) 0.1%, and (E, F) 0.05%.

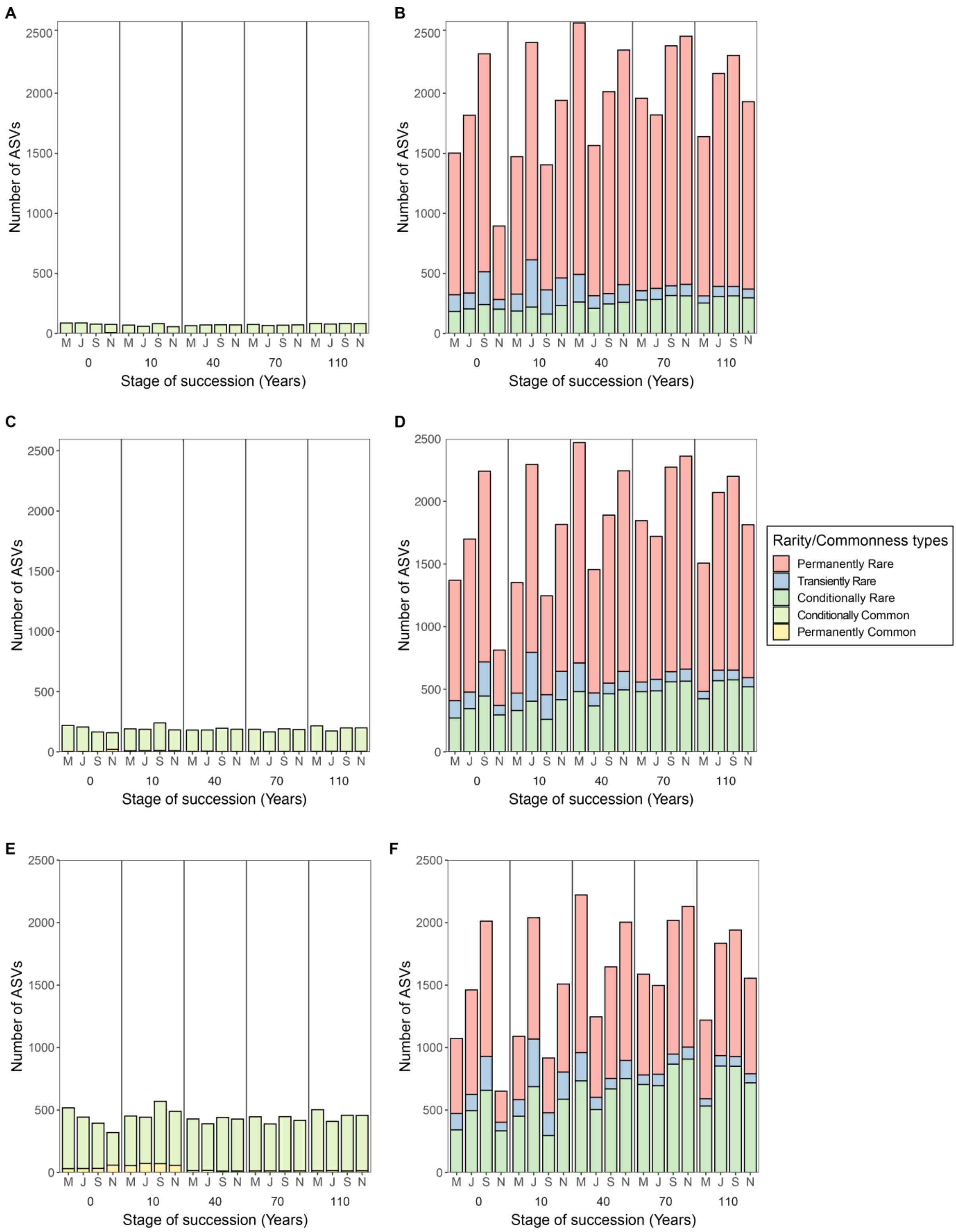

Figure 14 Bar charts displaying the number of ASVs of each type of commonness (A, C and E) and rarity (B, D and F). The plots display changes across five successional stages (0, 10, 40, 70 and 110 years) and four sampling times (M-May, J-July, S-September, N-November). The common and rare biospheres are shown at distinct rarity cutoffs: (A, B) 0.2%, (C, D) 0.1%, and (E, F) 0.05%.

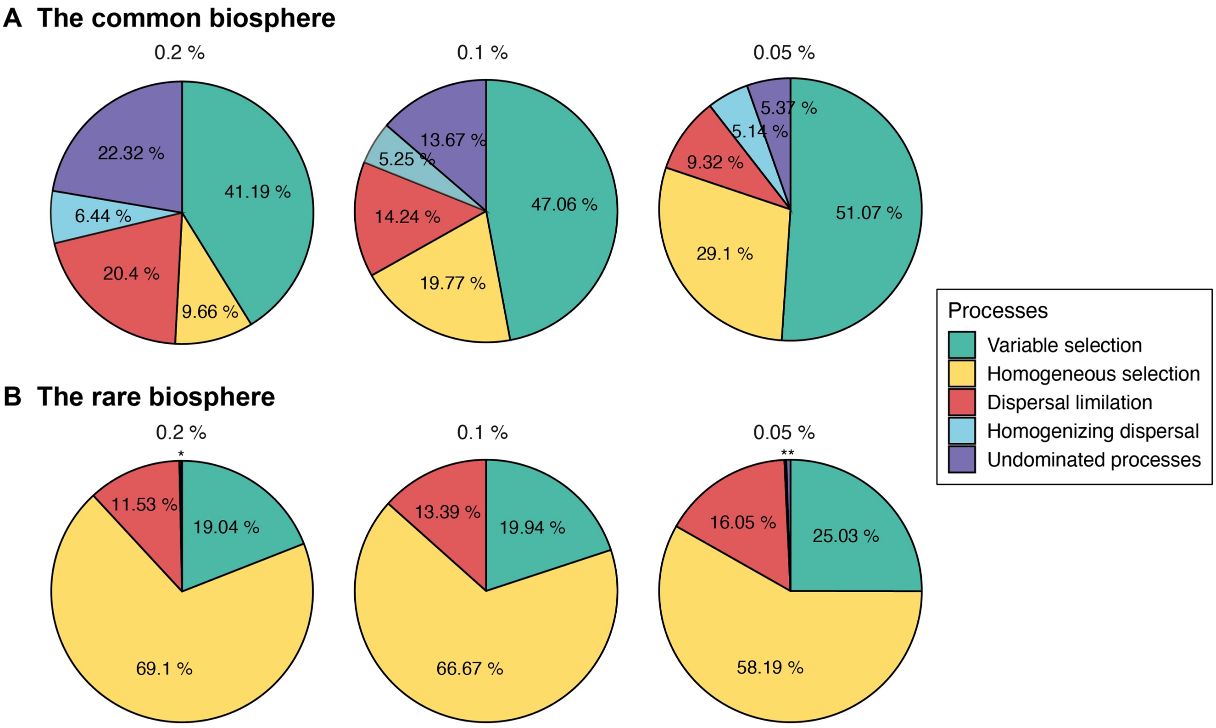

Figure 15 Plots displaying the relative influences of distinct assembly processes structuring the common (upper panel) and rare (lower panel) biospheres at different rarity cutoff values (0.2%, 0.1% and 0.05%). *indicates the relative influences of homogenizing dispersal (0.23%) and undominated processes (0.11%) for the rare biosphere at the rarity cutoff of 0.2%. **indicates the relative influences of homogenizing dispersal (0.23%) and undominated processes (0.51%) for the rare biosphere at the rarity cutoff of 0.05%.

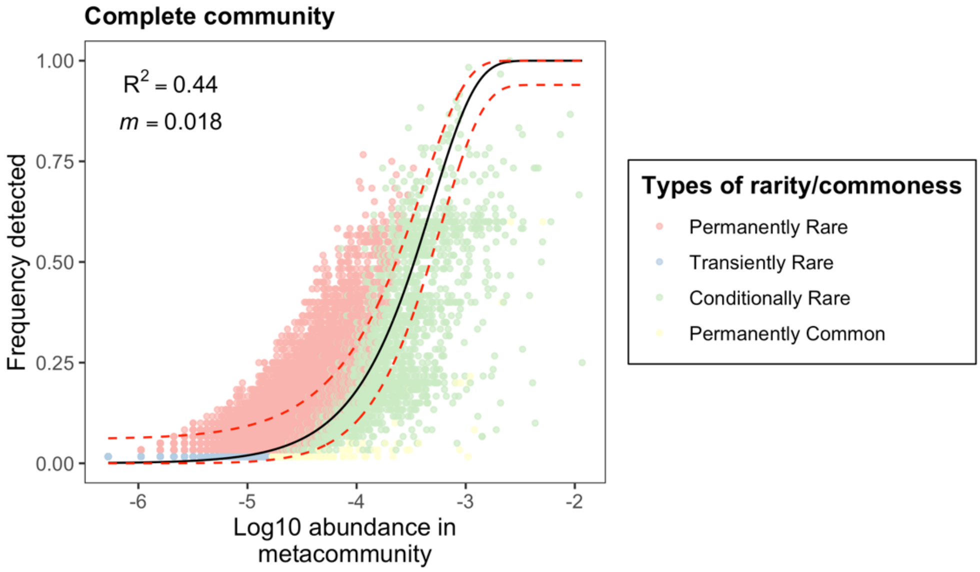

Figure 16 The scatter plots show the observed abundance-occurrence relationship from the Sloan's neutral model. The frequency of detected ASVs was plotted against their average relative abundance in the entire metacommunity. Dots represent different ASVs. The classification of ASVs to different types of rarity and commonness were shown in different colors. Solid lines present the model prediction of predicted occurrence of ASV at different abundance. Dashed lines represent 95% confidence intervals of the model. R^2 means the fit of the model, while m indicates the immigration rates, i.e., the possibility the space vacated by the death of an individual is filled by an immigrant.

Supplementary Tables

Table 1 Permutational multivariate analysis of variance (PERMANOVA) results showing the influence of successional stage (Year), sampling time (Month) and their interaction on the community β-diversity, based on (A) Jaccard distances and (B) Bray-Curtis distances, respectively.

	Df	Sums of Sqs	Mean Sqs	Pseudo-F	R^2	*Pr*(>F)
**(A) Jaccard**						
**Year**	4	10.40741	2.601853	14.87203	0.44966	1.00E-04
**Month**	3	1.311632	0.437211	2.49907	0.05667	2.00E-04
**Year:Month**	12	4.428039	0.369003	2.1092	0.191317	1.00E-04
**Residuals**	40	6.997974	0.174949		0.302353	
**Total**	59	23.14506			1	
**(B) Bray-Curtis**						
**Year**	4	12.31698	3.079246	32.22555	0.611171	1.00E-04
**Month**	3	0.997447	0.332482	3.479561	0.049493	2.00E-04
**Year:Month**	12	3.016551	0.251379	2.630784	0.149682	1.00E-04
**Residuals**	40	3.822118	0.095553		0.189654	
**Total**	59	20.1531			1	

Df - degrees of freedom; Sum of Sq - sum of squares; Mean Sqs - mean of squares; Pseudo-F - F value by permutation; R^2 - explained variation; *P*-values based on 9999 permutations

Table 2 Permutational multivariate analysis of variance (PERMANOVA) results based on Bray-Curtis distances of the bacterial rare and common biospheres. Results are shown at distinct rarity cutoff values: (A) 0.2%, (B) 0.1%, and (C) 0.05%.

	Df	Sums of Sqs	Mean Sqs	Pseudo-F	R^2	*Pr*(>F)
**(A) 0.2%**						
**Biosphere**	1	6.4850559	6.4850559	52.4431834	0.13034791	1.00E-04
**Month**	3	1.00684347	0.33561449	2.71403863	0.02023729	1.00E-04
**Year**	4	13.4257716	3.3564429	27.1427962	0.26985446	1.00E-04
**Biosphere:Month**	3	1.37958957	0.45986319	3.71880982	0.02772939	1.00E-04
**Biosphere:Year**	4	10.0923473	2.52308682	20.4036337	0.20285352	1.00E-04
**Month:Year**	12	2.88311531	0.24025961	1.94292523	0.05794986	1.00E-04
**Biosphere:Month:Year**	12	4.58647814	0.38220651	3.0908178	0.092187	1.00E-04
**Residuals**	80	9.89269603	0.1236587		0.19884058	
**Total**	119	49.7518973			1	
**(B) 0.1%**						
**Biosphere**	1	6.18931504	6.18931504	46.5730208	0.1249375	1.00E-04
**Month**	3	0.93622446	0.31207482	2.3482836	0.01889863	1.00E-04
**Year**	4	13.2813853	3.32034634	24.9847613	0.26809802	1.00E-04
**Biosphere:Month**	3	1.39579551	0.46526517	3.50100201	0.02817553	1.00E-04
**Biosphere:Year**	4	9.68026754	2.42006688	18.2103875	0.19540586	1.00E-04
**Month:Year**	12	2.75092876	0.22924406	1.72500325	0.05553024	1.00E-04
**Biosphere:Month:Year**	12	4.67378378	0.38948198	2.93075281	0.09434499	1.00E-04
**Residuals**	80	10.6315887	0.13289486		0.21460923	
**Total**	119	49.5392892			1	
**(C) 0.05%**						
**Biosphere**	1	5.98764676	5.98764676	38.1873866	0.11933411	1.00E-04
**Month**	3	0.97398009	0.32466003	2.07058275	0.01941147	3.00E-04
**Year**	4	12.4079705	3.10199262	19.7835638	0.24729149	1.00E-04
**Biosphere:Month**	3	1.34283206	0.44761069	2.85472457	0.02676271	1.00E-04
**Biosphere:Year**	4	9.27146817	2.31786704	14.7826498	0.18478084	1.00E-04
**Month:Year**	12	3.02840307	0.25236692	1.60951934	0.06035623	1.00E-04
**Biosphere:Month:Year**	12	4.61946812	0.38495568	2.45513001	0.09206624	1.00E-04
**Residuals**	80	12.5437162	0.15679645		0.24999691	
**Total**	119	50.175485			1	

Df - degrees of freedom; Sum of Sq - sum of squares; Mean Sqs - mean of squares; Pseudo-F - F value by permutation; R^2 - explained variation; *P*-values based on 9999 permutations

Table 3 Summary table displaying the percentage and richness of each type of commonness and rarity at distinct cutoff values (0.2%, 0.1%, and 0.05%).

**Rarity cutoff**	**Biosphere**	**Types of rarity/commonness**	**Proportion in each biosphere (relative abundance)**	**Number of ASVs in each biosphere**
**0.2%**	Rare	Permanently rare	66.92 ± 0.65%	1560.95 ± 59.27
		Transiently rare	3.33 ± 0.36%	139.88 ± 12.85
		Conditionally rare	29.75 ± 0.67%	249.25 ± 7.04
	Common	Conditionally common	98.54 ± 0.43%	73.88 ± 1.69
		Permanently common	1.46 ± 0.43%	0.98 ± 0.30
**0.1%**	Rare	Permanently rare	54.73 ± 0.85%	1258.00 ± 52.66
		Transiently rare	4.14 ± 0.42%	138.88 ± 12.78
		Conditionally rare	41.13 ± 0.96%	437.37 ± 14.85
	Common	Conditionally common	95.84 ± 0.41%	185.42 ± 4.06
		Permanently common	4.16 ± 0.41%	5.25 ± 0.69
**0.05%**	Rare	Permanently rare	40.96 ± 0.88%	815.42 ± 40.01
		Transiently rare	5.93 ± 0.58%	135.17 ± 12.41
		Conditionally rare	53.11 ± 1.13%	632 ± 27.24
	Common	Conditionally common	90.77 ± 0.66%	413.42 ± 8.62
		Permanently common	9.23 ± 0.66%	28.88 ± 2.88

References

1. Jia X, Dini-Andreote F, Salles JF. Comparing the influence of assembly processes governing bacterial community succession based on DNA and RNA data. Microorganisms. 2020;accepted.
2. Dini-Andreote F, Pylro VS, Baldrian P, van Elsas JD, Salles JF. Ecological succession reveals potential signatures of marine–terrestrial transition in salt marsh fungal communities. ISME J. 2016;10:1984–97.
3. Dini-Andreote F, Silva M, Triado-Margarit X, Casamayor EO, van Elsas JD, Salles JF. Dynamics of bacterial community succession in a salt marsh chronosequence: evidences for temporal niche partitioning. ISME J. 2014;8(10):1989-2001; doi: 10.1038/ismej.2014.54.
4. Caporaso JG, Lauber CL, Walters WA, Berg-Lyons D, Lozupone CA, Turnbaugh PJ, et al. Global patterns of 16S rRNA diversity at a depth of millions of sequences per sample. Proc Natl Acad Sci USA. 2011;108(Supplement 1):4516-22; doi: 10.1073/pnas.1000080107.
5. Caporaso JG, Lauber CL, Walters WA, Berg-Lyons D, Huntley J, Fierer N, et al. Ultra-high-throughput microbial community analysis on the Illumina HiSeq and MiSeq platforms. ISME J. 2012;6(8):1621-4.
6. Stegen JC, Lin X, Fredrickson JK, Chen X, Kennedy DW, Murray CJ, et al. Quantifying community assembly processes and identifying features that impose them. ISME J. 2013;7(11):2069-79; doi: 10.1038/ismej.2013.93.
7. Stegen JC, Lin X, Fredrickson JK, Konopka AE. Estimating and mapping ecological processes influencing microbial community assembly. Front Microbiol. 2015;6:doi: 10.3389/fmicb.2015.00370; doi: 10.3389/fmicb.2015.00370.
8. Galand PE, Casamayor EO, Kirchman DL, Lovejoy C. Ecology of the rare microbial biosphere of the Arctic Ocean. Proc Natl Acad Sci USA. 2009;106(52):22427-32; doi: 10.1073/pnas.0908284106.
9. Reveillaud J, Maignien L, Murat Eren A, Huber JA, Apprill A, Sogin ML, et al. Host-specificity among abundant and rare taxa in the sponge microbiome. ISME J. 2014;8(6):1198-209; doi: 10.1038/ismej.2013.227.
10. Logares R, Audic S, Bass D, Bittner L, Boutte C, Christen R, et al. Patterns of rare and abundant marine microbial eukaryotes. Current biology : CB. 2014;24(8):813-21; doi: 10.1016/j.cub.2014.02.050.
11. Campbell BJ, Yu L, Heidelberg JF, Kirchman DL. Activity of abundant and rare bacteria in a coastal ocean. Proc Natl Acad Sci USA. 2011;108(31):12776-81; doi: 10.1073/pnas.1101405108.
